# Targeting pancreatic cancer with combinatorial treatment of CPI-613 and inhibitors of lactate metabolism

**DOI:** 10.1371/journal.pone.0266601

**Published:** 2022-04-22

**Authors:** Simone Kumstel, Tim Schreiber, Lea Goldstein, Jan Stenzel, Tobias Lindner, Markus Joksch, Xianbin Zhang, Edgar Heinz Uwe Wendt, Maria Schönrogge, Bernd Krause, Brigitte Vollmar, Dietmar Zechner

**Affiliations:** 1 Rudolf-Zenker-Institute of Experimental Surgery, University Medical Center, Rostock, Germany; 2 Core Facility Multimodal Small Animal Imaging, University Medical Center, Rostock, Germany; 3 Department of Nuclear Medicine, University Medical Center, Rostock, Germany; Lobachevsky University, RUSSIAN FEDERATION

## Abstract

Pancreatic cancer is the fourth leading cause of cancer death, with a 5-year survival rate of 10%. A stagnant high mortality rate over the last decades highlights the need for innovative therapeutic approaches. Pancreatic tumors pursue an altered metabolism in order to maintain energy generation under low nutrient influx and hypoxic conditions. Targeting these metabolic strategies might therefore be a reasonable therapeutic approach for pancreatic cancer. One promising agent is CPI- 613, a potent inhibitor of two enzymes of the tricarboxylic acid cycle. The present study evaluated the anti-cancerous efficacy of CPI-613 in combination with galloflavin, a lactate dehydrogenase inhibitor or with alpha-cyano-4-hydroxycinnamic acid, an inhibitor of monocarboxylate transporters. The efficacy of both combination therapies was tested *in vitro* on one human and two murine pancreatic cancer cell lines and *in vivo* in an orthotopic pancreatic cancer model. Tumor progression was evaluated by MRI and ^18^F-FDG PET-CT. Both combinatorial treatments demonstrated *in vitro* a significant inhibition of pancreatic cancer cell proliferation and induction of cell death. In contrast to the *in vitro* results, both combination therapies did not significantly reduce tumor growth *in vivo*. The *in vitro* results suggest that a combined inhibition of different metabolic pathways might be a promising approach for cancer therapy. However, the *in vivo* experiments indicate that applying a higher dosage or using other drugs targeting these metabolic pathways might be more promising.

## Introduction

Pancreatic cancer is the fourth leading cause of cancer death, with a 5-year survival rate of 10% [1]. The overall survival, for other entities such as breast, lung, and prostate cancer was greatly improved in the last decades by innovative therapies and new screening methods. However, the progress in treating pancreatic cancer was only minor as indicated by a stagnant high mortality rate [1, 2]. Pancreatic cancer is therefore expected to be the second leading cause of cancer death by the end of this decade [3]. For this reason, the development of new therapeutic methods is still inevitable.

Targeting cancer metabolism might be a possible therapeutic approach. Characteristic for pancreatic ductal adenocarcinoma is excessive desmoplasia, which consists of collagen and extracellular matrix proteins, produced by activated pancreatic stellar cells [4]. This desmoplastic reaction leads to restricted vascularization, lower nutrient influx and hypoxia [5]. In order to adapt to these difficult conditions, pancreatic ductal adenocarcinoma adjust their metabolism. In addition, oncogenic *Kras* and its downstream activated signaling pathways alter cell metabolism [6]. The *Kras* mutation is expressed in 90% of the pancreatic ductal adenocarcinoma. Under hypoxic but also normoxic conditions pancreatic cancer cells have an increased rate of glycolysis and lactate production. This is facilitated by a *Kras* driven higher expression of the enzyme lactate dehydrogenase [6]. The *Kras* mutation also results in a higher expression of glucose transporter GLUT-1 and different glycolytic enzymes to increase the glycolytic flux [6, 7]. Pancreatic cancer cells also exchange fuels, such as lactate between hypoxic and normoxic cells [8]. In order to maintain this lactate transport, pancreatic tumors express many monocarboxylate transporters (MCTs) [9, 10].

Many inhibitors were investigated in the last decades to target cancer cell metabolism. One of them is CPI-613 (CPI), also known as Devimistat. CPI is an lipoic acid derivative and a potent inhibitor of enzymes of the tricarboxylic acid (TCA) cycle, such as pyruvate dehydrogenase (PDH) and α-ketoglutarate dehydrogenase (α-KGDH) [11, 12]. CPI demonstrated an effective growth inhibition in various pancreatic cancer cell lines and *in vivo* models [11–14]. The efficacy of CPI in combination with FOLFIRINOX was already tested in a phase 1 clinical study for patients with metastatic pancreatic cancer, the overall objective response rate with the maximum dose was 61%, with an overall median survival of 19 months. This treatment proved to be more efficient than the FOLFIRINOX therapy alone with an objective response rate of 31% and a median overall survival of 11.1 months [15]. According to these promising results CPI in combination with FOLFIRINOX is currently tested in a multicenter open label, international, Phase 3 randomized trial [16]. CPI might therefore be an important therapeutic agent to treat pancreatic cancer in the future. The main effect of CPI is the inhibition of the TCA cycle, which leads to reduced energy production, stimulation of distinct mitochondrial pathways and generation of reactive oxygen species (ROS) [11, 12]. Since pancreatic cancer cells are not relying on the TCA cycle but also use glycolysis for energy generation, the combinatorial treatment of CPI with inhibitors of the lactate metabolism could be a promising approach, to block the tumor-specific energy generation more efficiently. Therefore, the present study, focused on the combination of CPI, either with galloflavin, an inhibitor of the enzyme lactate dehydrogenase (LDH) [17], or alpha-cyano-4-hydroxycinnamic acid (CHC), a non-specific inhibitor of monocarboxylate transporters (MCTs), which are able to transport lactate across the cellular membrane [18–22]. The efficacy of both combinatorial treatments was tested *in vitro* and *in vivo* in a murine orthotopic pancreatic cancer model.

## Material and methods

### Reagents and antibodies

Dimethyl sulfoxide (DMSO), the primary antibody for β-actin (A5441) and the secondary antibody anti-mouse IgG HRP-linked (9044) were purchased from Sigma-Aldrich (St. Louis, USA). The metabolic inhibitors CHC and galloflavin were ordered from Tocris Bioscience (Bristol, UK). CPI-613 was bought from Hölzel Diagnostika (Cologne, Germany). Primary antibodies for cleaved caspase-3 (#9661), PARP (#9524), and secondary antibodies anti-rabbit IgG, HRP-linked (#7024) were purchased from Cell Signaling Technology (Danvers, Massachusetts, USA). The antibody for PDH (9H9AF5) was purchased from Thermo Fischer Scientific (Waltham, Massachusetts, USA).

### Cell culture

The 6606PDA cells, containing a *Kras* G12D mutation (a gift from Prof. Tuveson, University of Cambridge, UK) [23], were cultured in Dulbecco’s Modified Eagle’s Medium (DMEM, 4.5 g/l Glucose, Biochrom GmbH, Berlin, Germany), supplemented with 10% fetal calf serum (FCS), 1 ml/l Tylosine (8 mg/ml, Sigma-Aldrich) and 1 ml/l Amphotericin B (250 μg/ml, Biochrom GmbH). The national cancer institute provided the murine Panc02 cells. The human Mia Paca-2 cells were purchased from ATCC (Manassas, VA, USA). These two cell lines were cultured in RPMI 1640 medium (Sigma-Aldrich), supplemented with 10% FCS, penicillin and streptomycin.

### Analysis of proliferation, cell death and α-ketoglutarate-hydrogenase activity

To analyze the effect of distinct metabolic inhibitors on proliferation of the pancreatic cancer cells, 2–8 x 10^3^ cells per well were seeded in a 96-well plate for 24 h. The cells were then treated with the indicated concentrations of CPI, galloflavin, CHC, combinations or appropriate solvents (CPI: 100% DMSO, CHC: 100% DMSO, galloflavin: 50% DMSO/ 50% PBS) in control groups. The proliferation of 6606PDA cells was further quantified by incorporation of 5-bromo-2’-deoxyuridine (BrdU) with colorimetric Cell Proliferation ELISA kit (Roche Diagnostics, Mannheim, Germany) and absorbance was measured with the Perkin Elmer Victor X3 model 2030 Multilabel Plate Reader (PerkinElmer, Waltham, USA). The absorbance for each treatment was calculated by the mean value of three technical replicates and all experiments were repeated independently as indicated in figure legends. To assess the effect of the therapies on cell death 1.5–3 x 10^4^ pancreatic cancer cells were cultured in a 24-well plate and were treated with indicated concentrations and durations respectively for each combinatorial treatment. The percentage of cell death was quantified after incubating cells for the indicated durations with trypan blue solution. 50 cells were counted manually for two times in a blinded fashion and the cell death was calculated. If a difference of more than 30% was quantified, the cells were counted for a third time and the mean value was estimated. The *in vitro* quantification of α-ketoglutarate dehydrogenase activity was performed with the colorimetric assay kit (#K678-100) from BioVision Inc. (Milpitas, USA). For this purpose, 3 x 10^5^ 6606PDA cells were plated per well in a 6-well plate and incubated with 300 μM CPI and the respective DMSO concentration for 4 h. The cells were washed with PBS and homogenized in 100 μl ice cold ketoglutarate dehydrogenase assay puffer. Afterwards the assay was performed according to the manufactures instructions.

### Western blot

To analyze apoptosis and pyruvate dehydrogenase (PDH) expression, 1.5–3 x 10^5^ 6606PDA cells were plated in a 6-well plate, cultured for 24 h and treated with the indicated concentrations of the metabolic inhibitors and the respective solvents for the indicated time periods. The proteins (for apoptosis: 25–40 μg, for PDH: 10 μg) of the cell lysates were further separated by SDS polyacryl gels and transferred to a polyvinyl difluoride membrane (Immobilon-P; Millipore, Eschborn, Germany), as previously described [24]. To quantify apoptosis the membranes were blocked with 2.5% BSA and incubated overnight at 4°C with primary antibody rabbit anti-cleaved caspase-3 (dilution: 1000x, #9661, Cell Signaling Technology) or rabbit anti-PARP (dilution 1000X, #9524, Cell Signaling Technology), followed by peroxidase-linked secondary anti-rabbit antibody (dilution: 20000x, #7024, Cell Signaling Technology). For analysis of the housekeeping protein, membranes were stripped, blocked with 2.5% BSA and incubated with mouse anti-β-actin antibody (dilution: 20000×, A5441, Sigma-Aldrich) followed by peroxidase-linked anti-mouse antibody (dilution: 60000×, 9044 Sigma-Aldrich). The protein expression of pyruvate dehydrogenase was quantified by incubating the membranes over night at 4°C with primary antibody for PDH (dilution: 1000x, 9H9AF5, Thermo Fischer Scientific), followed by peroxidase linked anti-mouse antibody, 9044 Sigma-Aldrich (dilution: 60000x). Protein expression was visualized by luminol-enhanced chemiluminescence (ECL plus; GE Healthcare, Munich, Germany) and digitalized with Chemi-Doc XRS System (Bio-Rad Laboratories, Munich, Germany).

### Animals

C57BL6/J mice were originally purchased from the Jackson Laboratory (Bar Harbor, Main, USA) and bred in our animal facility under specified pathogen free (SPF) conditions (the following pathogens were detected within the last two years: *Helicobacter* sp., *Rodentibacter pneumotropicus*, *murine Norovirus* and *rat Theilovirus*). During the experiment 14–24 weeks old male C57BL6/J mice were housed separately in type III cages (with a 12h / 12h dark / light cycle) with food and water *ad libitum*. Enrichment was provided by nesting material, paper roles and wooden sticks. All animal experiments were approved by the local authority (Landesamt für Landwirtschaft, Lebensmittelsicherheit und Fischerei Mecklenburg-Vorpommern, Az. 7221.3-1-019/15; 7221.3-1-062/16). These decisions were in accordance with the protection of animal act for Germany and the European Directive 2010/63/EU.

### The orthotopic pancreatic cancer model and therapeutic treatment of mice

The orthotopic injection of tumor cells was performed as previously described [25, 26]. The mice were anaesthetized with 1–3 vol. % isoflurane. For perioperative analgesia, carprofen (5 mg/kg) was injected subcutaneously and eye ointment was applied. During surgery, the mice were kept warm by a heating plate. The abdomen of mice was shaved, opened and the 6606PDA cells (2.5 x 10^5^ cells in 5 μl PBS/Matrigel) were injected with a 25 μl syringe (Hamilton, Reno, Nev., USA) into the head of the pancreas. Afterwards the abdomen was closed with two sutures (Johnson & Johnson MEDICAL GmbH, New Brunswick, USA) and mice were placed in front of a heating lamp for 20–30 min. 1250 mg/l metamizol was added daily to the drinking water of mice for continued analgesia until end of the experiment. After 4 days of recovery, mice were assigned into the different treatment groups, matching the age. All therapeutics were injected intraperitoneally from day 4 until euthanasia of mice at day 37, the drug concentrations and application intervals were performed as indicated in the figures. The drugs were either solved in DMSO (Dimethylsulfoxide, Sigma-Aldrich; CPI: 100% DMSO, CHC: 50% DMSO, 50% PBS, galloflavin: 100% DMSO) or 30% PEG 300 (Polyethyleneglycol 300, Sigma-Aldrich), 1% Tween 80 and PBS. Sham treatment was performed with the appropriate solvent for each drug. On day 37 after tumor cell injection the mice were euthanized by cervical dislocation in deep narcosis (by using 4–5 vol. % isoflurane or i.p. injection of 98 mg/kg Ketamine and 6,5 mg/kg Xylazin) and the tumors were extracted and weights of tumors were assessed. The body weight of mice was assessed at the indicated days after tumor cell injection, to allow a constant monitoring of the health status. The body weight change in % was calculated from the individual body weight of mice assessed at the beginning of the experiment before tumor cell injection. We used 59 mice in total for all experiments, 10 mice had to be euthanized during the experiment, when reaching humane endpoint criteria, such as body weight loss of >20% or apathy. 9 mice reached humane endpoint criteria within 1–3 days after tumor cell injection. One sham treated animal, from the first CPI+CHC experiment with DMSO as solvent, had to be euthanized 21 days after tumor cell injection, due to apathy.

### MRI and ^18^F-FDG PET-CT imaging

For quantification of tumor progression *in vivo*, mice were scanned at the indicated days with a 7T MRI (magnetic resonance imaging, BioSpec 70/30, 7.0 Tesla, gradient insert: BGA-12S, Bruker BioSpin GmbH, Ettlingen, Germany), combined with a transmit volume resonator (86 mm inner diameter) and a receive surface coil, as described previously [27]. Animals were anesthetized with 1.0–2.5 vol.% isoflurane and were scanned with morphological T2 weighted TurboRARE (Rapid Acquisition with Relaxation Enhancement) sequences with following parameters: TE/TR: 25/1880 ms; FoV: approx. 40 x 28 mm; matrix: 200 x 200; voxel size: 0.2 x 0.14 mm, slice thickness 1 mm, 25 slices. Tumor volume was further quantified with the program 3DSlicer (version 4.4.0, www.sclicer.org). Additional to the MRI, also PET-CT imaging with the tracer ^18^F-FDG was performed on mice undergoing CPI + CHC treatment and the respective sham group on day 32 after tumor cell injection. As described before [27], mice were injected intravenously with ~15MBq of ^18^F-FDG in the tail vain under isoflurane anesthesia. 60 minutes after injection static PET scans in headprone position were recorded for 15 minutes (Inveon PET-CT Siemens, Knoxville, TN, USA). Through all imaging procedures, the body temperature was monitored and kept constant by a heating pad. The PET image reconstruction method contained a 2-dimensional ordered subset expectation maximization (2D-OSEM) algorithm with four iterations and six subsets. Attenuation correction was achieved with the whole body CT scan and a decay correction for ^18^F was applied. PET images were further adjusted for random coincidences, dead time and scatter. The ^18^F-FDG uptake (% injected dose / g bodyweight) in each tumor was quantified as metabolic tumor volume (30% of the hottest voxel) [28] with the program Inveon Research Workplace (Siemens Healthcare AG, Zurich, Switzerland).

### Immunohistochemistry

The tumor tissue was fixed with 4% paraformaldehyde, sliced in 4 μm sections and blocked with protein block serum-free (X0909, Dako, Jena, Germany). The expression of the metabolic targets was analyzed by immunohistochemistry using anti-rabbit PDH E1 alpha polyclonal antibody (18068-1-AP, dilution 1:500, Proteintech, Rosemont, IL, USA), anti-mouse monoclonal antibody OGDH (66285-1-Ig, dilution 1:100, Proteintech), anti-rabbit LDHA polyclonal antibody (19987-1-AP, dilution 1:100, Proteintech), anti-rabbit anti-MCT-4 antibody (bs-2698R, dilution 1:100, Biossusa, Woburn, MA, USA). Goat anti-rabbit Immunoglobulins/HRP (P0448, Dako) or goat anti-mouse Ig/HRP antibody (P40447, Dako) were used as secondary antibodies.

### Data and statistics

Data was graphed and analyzed, either with the program SigmaPlot 12.0 (SYSTAT Software Inc., San Jose, USA), or the program GraphPad Prism 8.0 (GraphPad Software, San Diego, USA). Medication dose-response curves are presented as mean value ± standard deviation. Data is either graphed in box blots or bar graph, the 10^th^ and 90^th^ percentile as whiskers, or presented as median ± 95% confidence interval. Significant differences were evaluated either, by ANOVA on Ranks, or by Two Way repeated measure ANOVA and for correction of multi-comparison by Sidak’s test, p < 0.05 was considered to be significant.

## Results

### CPI combined with CHC inhibited cancer cell proliferation and induced apoptosis

**Fig 1:**
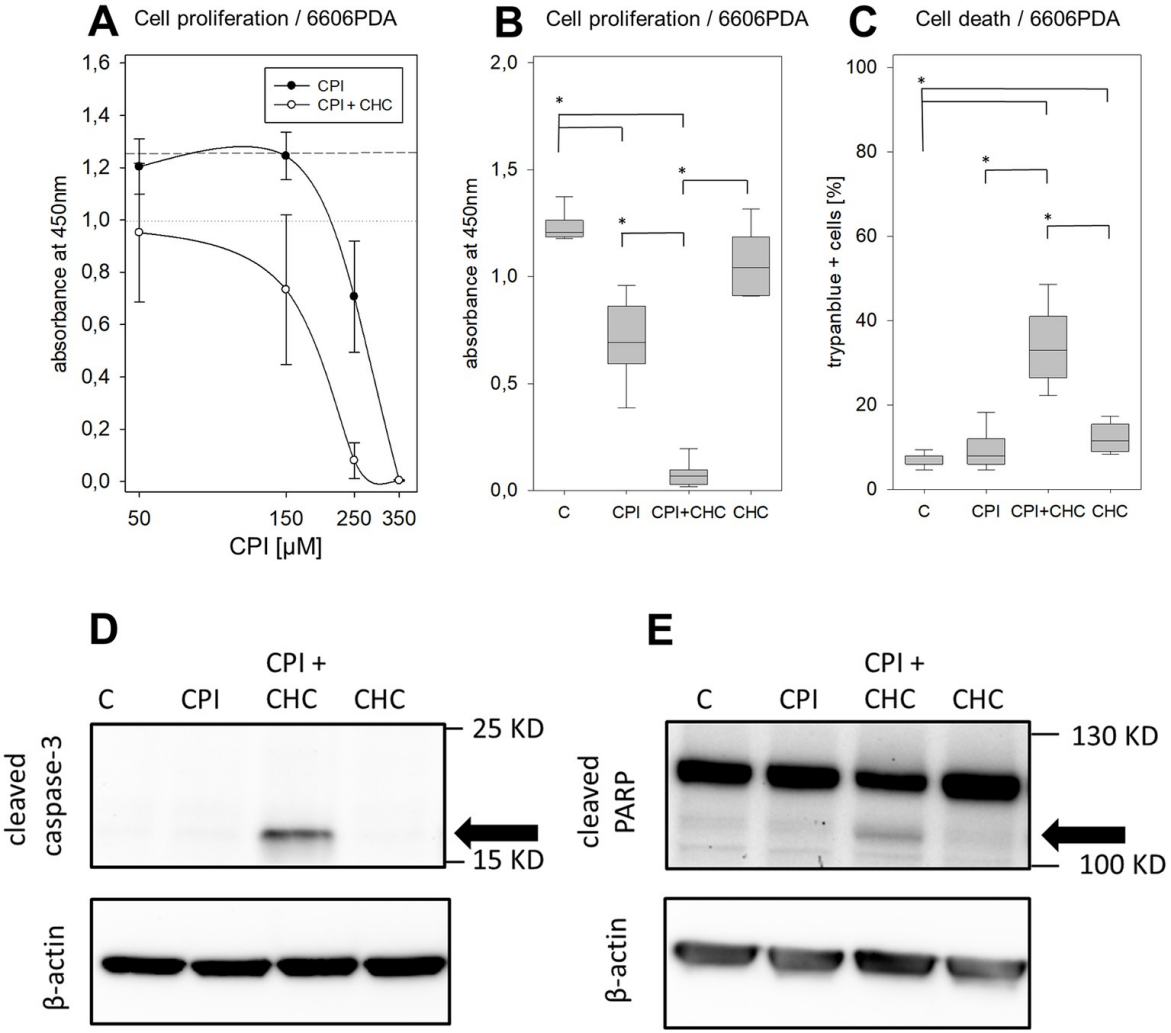
CPI-613 in combination with CHC leads to a reduction in pancreatic cancer cell proliferation and viability. (**A**) Quantification of proliferation by BrdU-ELISA, dose-response curve after 48 h treatment of 6606PDA cells with CPI-613 (50–350 μM), with and without 5 mM CHC (dotted lines indicate mean value of absorbance for control treated cells, either with high or low DMSO concentrations). (**B**) Evaluation of cell proliferation with vehicle control (medium with the respective amount of vehicle, DMSO) (C), 250 μM CPI-613 (CPI), 5 mM CHC (CHC) and the combination (CPI + CHC). (**C**) Evaluation of cell death by trypan blue assay after cultivating 6606PDA cells for 48 h with vehicle control with DMSO (C), or medium supplemented with 150 μM CPI-613 (CPI), 5 mM CHC and CPI-613 plus CHC (CPI + CHC). (**D**) Apoptosis was evaluated by quantifying cleavage of caspase 3 (caspase-3) and PARP (**E**) via western blot, after treating the cells 24 h with DMSO only (C), 150 μM CPI-613, 5 mM CHC or the combination (the bands of the cleaved proteins are indicated by black arrows). Statistics were performed with ANOVA on Ranks and correction for multi comparison by Holm-Sidak method, significant differences: * p ≤ 0.002; Independent experiments: A-B: n = 5–7, C: n = 7, D-E: n = 3.

CPI monotherapy caused a dose dependent inhibition of pancreatic cancer cell proliferation. After treating the 6606PDA cells for 48 h with CPI the IC_50_ value was estimated at 254 μM. A stronger inhibition of BrdU incorporation was observed when applying 5 mM CHC in addition to CPI (Fig 1A). 250 μM CPI in combination with 5 mM CHC (median: 0.07, IQR: 0.02–0.10, absorbance at 450 nm) was able to significantly inhibit the cell proliferation compared to control treatment (median: 1.21; IQR: 1.18–1.30) with the respective concentration of DMSO and the monotherapies of either CPI (median: 0.69, IQR: 0.53–0.89), or CHC (median: 1.04, IQR: 0.91–1.22, Fig 1B). A significant induction of cell death was induced with 150 μM CPI plus 5 mM CHC (median: 33.00%, IQR: 25.75–42.00%), when compared to control treatment (median: 6.00%, IQR: 6.00–8.00%) or the CPI (median: 8.00%, IQR: 6.00–12.00%) and CHC monotherapy (median: 11.50%, IQR: 9.00–15.75%, Fig 1C). This combination of drugs induced features of apoptosis, such as the cleavage of caspase-3 and PARP (Fig 1D and 1E). Thus, the CPI plus CHC combinatorial therapy had a strong anti-cancerous effect *in vitro*. Similar results were obtained when using one additional murine and one humane pancreatic cancer cell line (S1 Fig).

### Impairment of proliferation and survival of pancreatic cancer cells by CPI in combination with galloflavin

**Fig 2:**
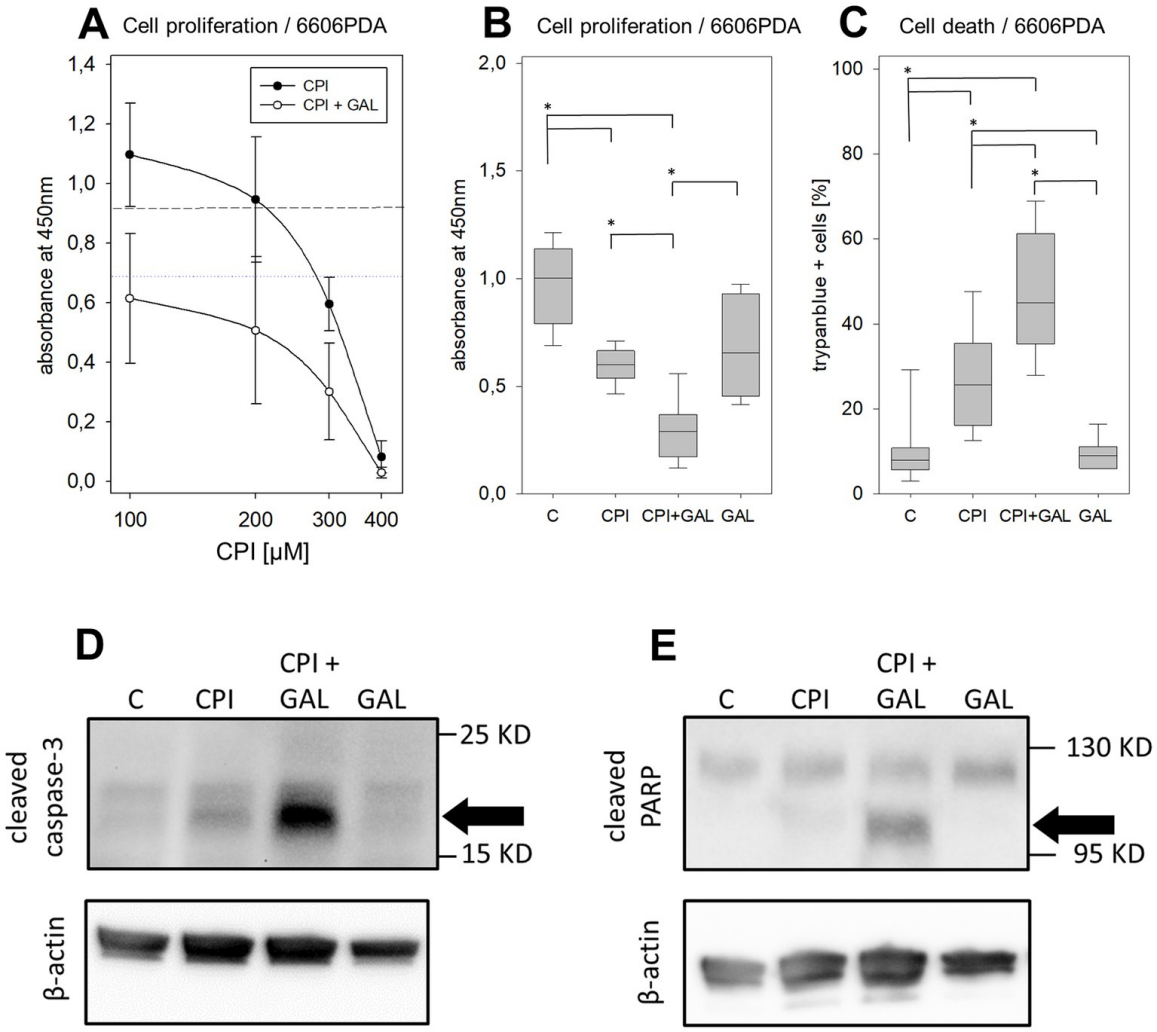
CPI-613 in combination with galloflavin inhibits pancreatic cancer cell proliferation and induces cell death. (**A**) Proliferation of 6606PDA cells after cultivating them in CPI-613 (100–400 μM) with and without 30 μM galloflavin (GAL; dotted lines indicate mean value of absorbance for control treated cells, either with high or low DMSO concentrations). (**B**) Analysis of proliferation by BrdU-ELISA, (**C**) or quantification of cell death by trypan blue assay, after cultivating 6606PDA cells 24 h in control medium with the respective amount of the vehicle DMSO (C), medium supplemented with 300 μM CPI-613 (CPI), 30 μM galloflavin (GAL) or the combination of both drugs (CPI + GAL). (**D-E**) Apoptosis was assessed by analyzing the cleavage of caspase 3 (caspase-3) and PARP via western blot, after treating the cells with medium supplemented with DMSO (C), 300 μM CPI-613, 30 μM galloflavin or the combination for 24 h respectively (the bands of the cleaved proteins are indicated by black arrows). Statistics were performed with ANOVA on Ranks Holm-Sidak method for multi comparison, significant differences: * p ≤ 0.004; Independent experiments: A-B: n = 8–10, C: n = 19, D-E: n = 2–3.

The efficacy of the combinatorial treatments was tested on murine pancreatic cancer cells (6606PDA) *in vitro*. CPI was able to inhibit the pancreatic cancer cell proliferation in a dose dependent manner. The addition of 30 μM galloflavin enhances this inhibitory effect (Fig 2A). The IC_50_ concentration of CPI after 24 h treatment was calculated at 316 μM. 300 μM CPI in combination with 30 μM galloflavin (median: 0.29, IQR: 0.17–0.40 absorbance at 450 nm) induced a significant reduction of cancer cell proliferation compared to the DMSO control (median: 1.00, IQR: 0.78–1.15) and to both monotherapies (CPI: median: 0.60, IQR: 0.53–0.67; Gal: median: 0.66, IQR: 0.44–0.93, Fig 2B). By using the same concentrations, a significant induction of cell death was observed in the combination therapy (median: 45.00%, IQR: 35.00–61.67%) compared to the DMSO control (median: 7.30%, IQR: 5.00–9.33%) and the monotherapies (CPI: median: 25.67%, IQR: 16.00–36.67; Gal: median: 9.00, IQR: 6.00–11.33%, Fig 2C). CPI combined with galloflavin caused a stronger induction of apoptosis, compared to control and each monotherapy as indicated by the protein cleavage of caspase-3 and PARP (Fig 2D and 2E). Thus, a strong anti-cancerous effect was observed in 6606PDA cells by this drug combination. However, a weaker combinatorial effect was found in one additional murine and one humane pancreatic cancer cell line (S2 Fig).

### CPI plus CHC treatment did not cause a significant reduction of tumor growth *in vivo*

**Fig 3:**
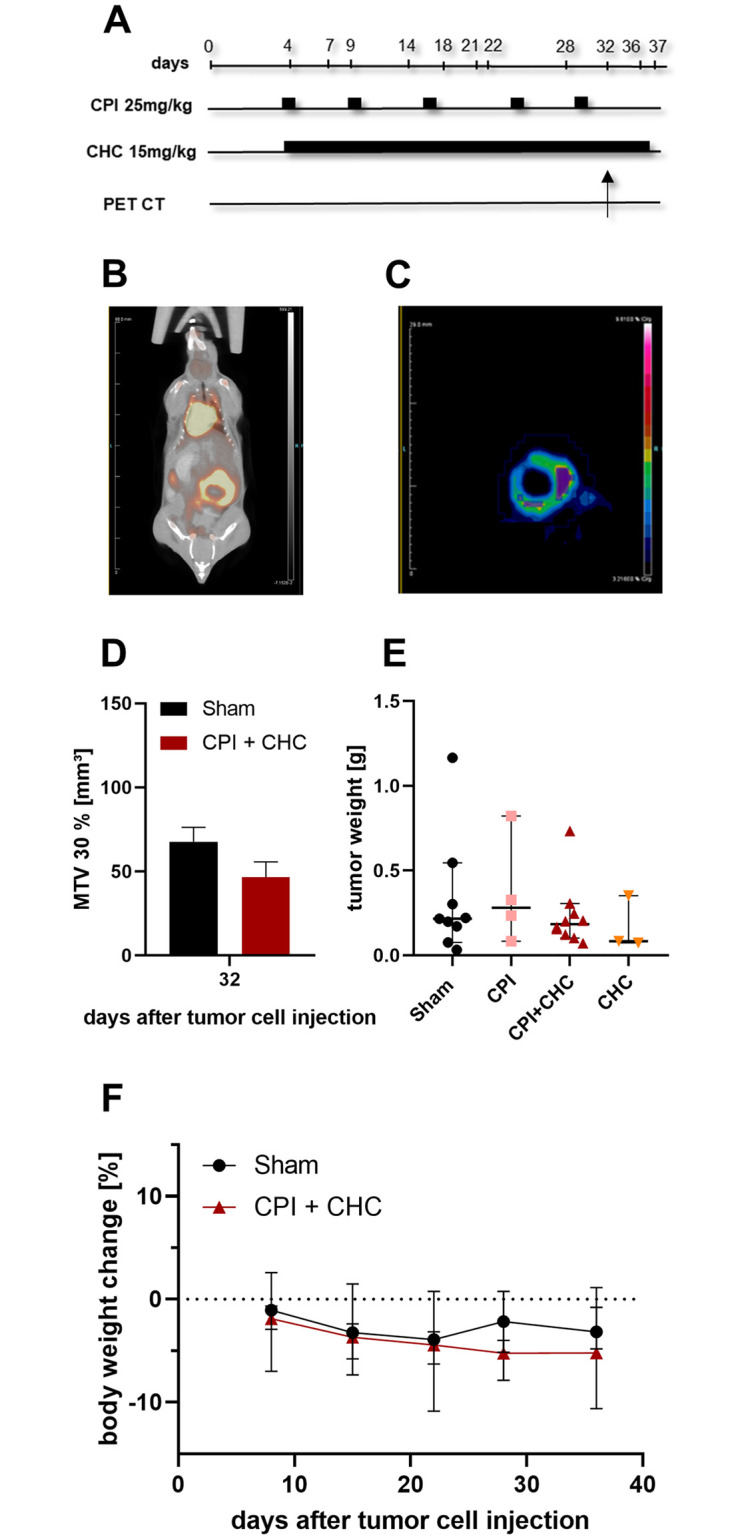
The combination therapy of CPI-613 and CHC (solvent: DMSO) did not lead to a significant reduction of tumor growth *in vivo*. (**A**) To evaluate the efficacy of the combinatorial therapy in the orthotopic pancreatic cancer model CPI-613 (25 mg/kg) was injected once a week and CHC (15 mg/kg) was applied on a daily basis from day 4 until the end of the experiment day 37. Both therapeutics were dissolved in DMSO (CPI: 100% DMSO, CHC: 50% DMSO/ 50% PBS), the sham animals were treated with the respective vehicle of the combinatorial treatment. (**B-C**) PET-CT imaging with the tracer ^18^F-FDG was performed to quantify changes in the glucose metabolism of tumor tissue. (**D**) The uptake of ^18^F-FDG in the tumor tissue was evaluated by metabolic tumor volume (MTV, tumor volume [mm³] with the criteria 30% of the hottest voxel of injected dose/g bodyweight) on day 32 after tumor cell implantation on a subset of sham and CPI + CHC tumors. (**E**) Tumor weight was measured at the end of the experiment in the indicated treatment groups. (**F**) The body weight of mice was measured at the indicated days during the treatment period and the body weight change was calculated from the weight assessed at the beginning of each experiment. Biological replicates: D: Sham n = 3, CPI + CHC n = 2. E-F: Sham n = 9, CPI n = 4, CPI + CHC n = 10, CHC n = 3.

A possible anticancer effect of the combinatorial treatment CPI plus CHC was also tested *in vivo*, by using an orthotopic pancreatic cancer mouse model. Treatment was conducted by i.p. injection of CPI (25 mg/kg, once a week) and CHC (15 mg/kg, daily) from day 4 until day 37. To evaluate the metabolic active tissue of the tumors PET CT imaging was performed on day 32 after tumor cell injection (Fig 3A), as indicated by exemplary images of ^18^F-FDG uptake (% injected dose / g bodyweight, Fig 3B) and quantified metabolic tumor volume (MTV, 30%, Fig 3C). No significant differences of MTV (30%) were observed in the combinatorial treatment group and the sham treated tumors (Fig 3D). When comparing the tumor weight at the end of the experiment no anti-cancer effects could be observed for the CPI + CHC treatment (Fig 3E). No significant differences of the body weight change were observed between sham and the combinatorial treatment group (Fig 3F).

**Fig 4:**
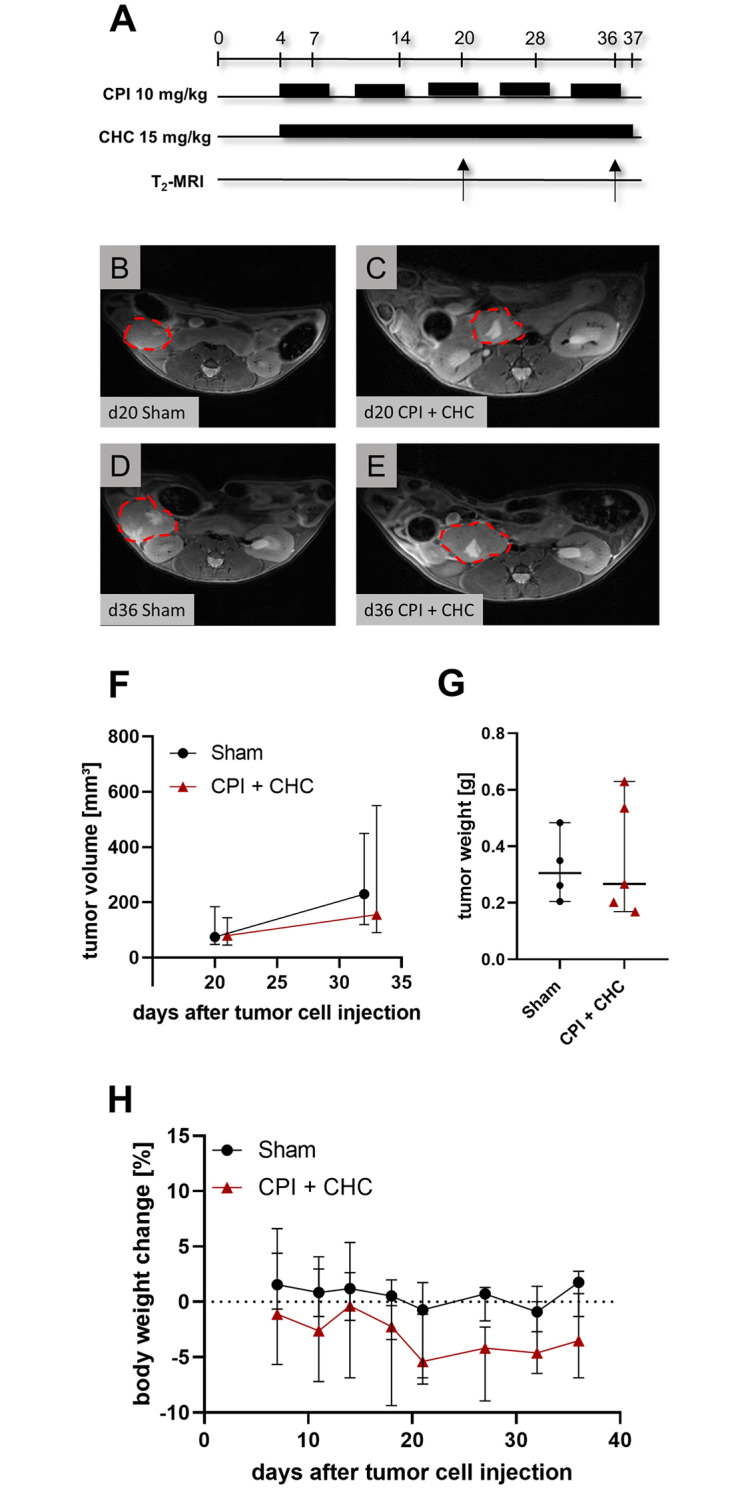
The combinatorial treatment of CPI + CHC (solvent: 30% PEG 300) did not inhibit the tumor growth *in vivo*. (**A**) CPI was injected 5x weekly at a dose from 10 mg/kg and CHC (15 mg/kg) was applied daily, from day 4 until day 37 after tumor cell injection. Both therapeutics were dissolved in 30% PEG 300 (30% Polyethylene glycol 300), 1% Tween, PBS. (**B-E**) MRI was performed on day 20 and 36 after tumor cell injection, as indicated by exemplary images (tumors are framed by a red dotted line). (**F**) The tumor volume was evaluated for the combinatorial treatment (CPI + CHC) and the sham treatment. (**G**) The weight of the tumors was quantified after sacrificing the mice on day 37. (**H**) The body weight change of mice quantified at the indicated days during the treatment period. Biological replicates: F-H: Sham: n = 4, CPI + CHC: n = 5.

In order to improve the *in vivo* response of the CPI + CHC therapy another experiment was performed. CPI was therefore injected more frequently (5x weekly) with a concentration of 10 mg/kg and CHC was applied daily from day 4 until day 37 (Fig 4A). Instead of DMSO we used PEG 300 (30%), in combination with Tween (1%) and PBS as solvent for both therapeutics, since DMSO was reported to be toxic in high concentrations and volume [29] and might even have anti-cancerous effects [30]. MRI was performed on day 20 and 36 to analyze the tumor progression *in vivo*, as indicated by exemplary images of sham and CPI + CHC treated tumors (Fig 4B–4E). No significant difference between control and combinatorial treatment was observed in the tumor volume assessed by MRI (Fig 4F). In addition, the tumor weight, assessed on day 37 did not indicate significant differences between the treatment groups (Fig 4G). No significant differences of body weight change were observed between sham and CPI + CHC treated mice, when using PEG 300 as solvent (Fig 4H).

### No anticancer efficacy of CPI and galloflavin in a murine orthotopic pancreatic cancer model

**Fig 5:**
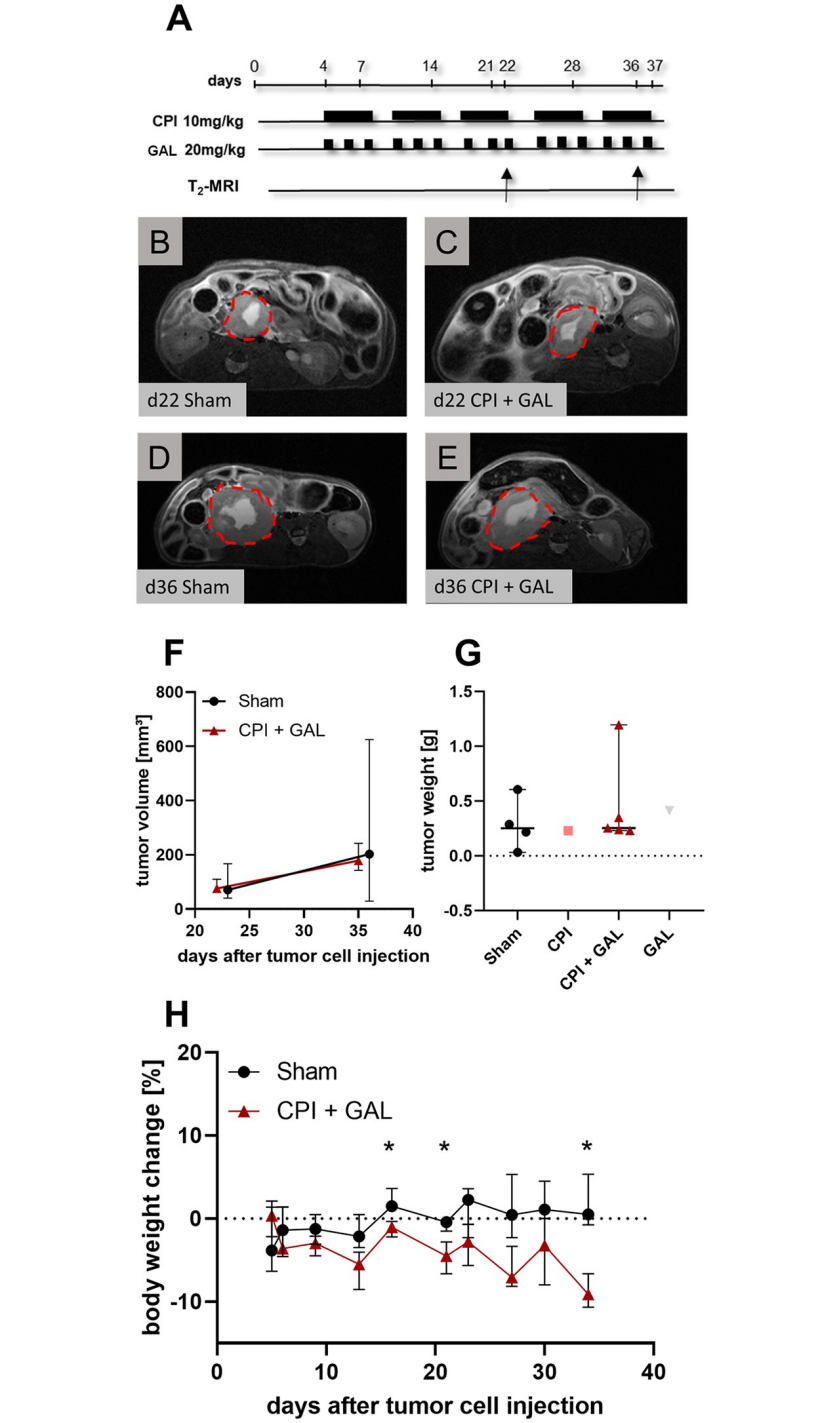
The combinatorial treatment of CPI-613 and galloflavin did not cause an impairment of the tumor growth *in vivo*. (**A**) To quantify the therapeutic effect *in vivo*, the 6606PDA cells were injected at day 0 into the pancreas of C57BL6/J mice. The treatment started after 4 days of recovery, CPI (10 mg/kg) was injected five times per week and galloflavin (GAL, 20 mg/kg) three times weekly. (**B-F**) The tumor of the combinatorial therapy and sham treated mice was identified by MRI on day 22 and 36 (tumors are framed by a red-dotted line) and the tumor volume was quantified at these time points. (**G**) The tumor weight of all treatment groups was measured at the end of the experiment on day 37. (**H**) The body weight change of sham and CPI + galloflavin treated mice was quantified at the indicated days. Biological replicates: F: n = 4 per group; G-H: Sham n = 4, CPI n = 1, CPI-GAL n = 5, GAL n = 1. *P < 0.05 was considered significant, calculated by Two-Way repeated measure ANOVA and correction of multi-comparison by Sidak’s method.

Based on the promising *in vitro* results of CPI in combination with galloflavin (GAL) a possible anticancer effect was explored *in vivo*. For this purpose, 6606PDA cells were implanted into the pancreas of C57BL6/J mice on day 0. Mice were treated with CPI (10 mg/kg, five times per week) and galloflavin (20 mg/kg, three times per week) from day 4 until day 37 (Fig 5A). Tumor progression was quantified by MRI on the days 22 and 36, as indicated by exemplary MRI images of sham and CPI + galloflavin treated tumors (Fig 5B–5E). However, no impairment on the tumor growth was observed with CPI + galloflavin treatment (Fig 5F). In addition, no anti-cancer effect of the combinatorial treatment could be observed when analyzing tumor weight at the end of the experiment (Fig 5G). CPI + galloflavin treated mice had a significant lower body weight change on day 16 (median: -1.04%, IQR: -2.20 –-0.37%), 21 (median: -4.51%, IQR: -5.75 –-3.03%) and 34 (median: -9.13%, IQR: -10.43 –-7.62%) after tumor cell injection compared to sham treated mice (day 16 median: 1.51%, IQR: 1.13–3.17%; day 21 median: -0.43%, IQR:- 1.26 –-0.10); day 34 median: 0.49%, IQR: -0.67–4.35% Fig 5H).

## Discussion

The present study demonstrated anti-proliferative and pro-apoptotic effects on pancreatic cancer cells for CPI in combination with CHC (Fig 1), or galloflavin (Fig 2). However, it is worth noticing, that the used *in vitro* concentrations (50–400 μM) and also the estimated IC_50_ values after 24 h/ 48 h treatment (6606PDA cells: 316/ 254 μM, Mia Paca cells: 296 μM/ 226 μM, Panc02 cells: 321/ 230 μM) were quite high for CPI-613. Similar dose-response curves and IC_50_ concentrations from 120–280 μM CPI (24-48h treatment), were also obtained in previous reported studies with human lung cancer, sarcoma [11] and pancreatic cancer cell lines [13].

CPI is reported in the literature to inhibit two enzymes of the TCA cycle, namely pyruvate dehydrogenase and α-ketoglutarate dehydrogenase. The protein expression of pyruvate dehydrogenase in 6606PDA cells was verified by western blot (S3A Fig). Zacher et al. reported that the substantial inhibition of the pyruvate dehydrogenase is caused by phosphorylation of its E1 α-subunit by CPI regulated pyruvate dehydrogenase kinases (PDKs) [11]. Interestingly, these PDKs are expressed exclusively in malignant tissue [31, 32]. In addition, CPI induces a redox–mediated inactivation of the enzyme α-ketoglutarate dehydrogenase on its E3 subunit [12]. We observed a 30% reduction of the α-ketoglutarate dehydrogenase activity in 6606PDA cells when treating them with CPI (S3B Fig).

CHC was reported to block the lactate transport by competitive inhibition of the MCT-1, MCT-2 and MCT-4 [33, 34]. Blocking the efflux for lactate inhibits the metabolic crosstalk of normoxic and hypoxic tumor cells [35]. An intracellular increase of lactate causes acidification of the cells and results in cell death [33]. Based on the proliferation data, we quantified an IC_50_ concentration for 48 h treatment of 6606PDA cells at 10 mM CHC in our previous study [33]. We observed previously on the 6606PDA cells that the anti-cancerous effect of CHC is not exclusively caused by the inhibition of lactate efflux, but might also be explained by an additional stimulation of the p38 signaling pathway [36].

Galloflavin is able to inhibit the isoforms A and B of LDH by directly binding to this enzyme without competing with the substrate [17]. This was also confirmed in 6606PDA cells, since we could report a significant inhibition of lactate concentration in the cell lysate and cell supernatant [37]. We could quantify an IC_50_ concentration of 102 μM galloflavin for 24 h treatment of 6606PDA cells in a previous study [37].

According to the above-mentioned literature, as well as our present and previous published results [36, 37], we suggest that the anti-proliferative and apoptotic effects, which we observed *in vitro*, are caused by inhibition of different metabolic aspects and signaling pathways.

Regarding the promising *in vitro* results, both combinatorial treatments were further quantified in a syngenic orthotopic pancreatic cancer model in mice. However, both combination therapies failed to significantly reduce the tumor progression *in vivo*, even when compared to sham treated mice (Figs 3 and 4). The expression of the metabolic targets PDH, α-KGDH, LDH and MCT-4 was checked by immunohistochemistry (S4 Fig). These proteins were expressed in the tumors, but their expression level was unaltered after therapeutic treatment. This is expected, because CPI, galloflavin and CHC do not primarily inhibit the expression but rather the activity of these proteins [11, 12, 17, 33, 34].

One reason for these inefficient anti-cancerous effects might be a potential chemoresistance *in vivo*. While the therapeutic efficacy *in vitro* was tested on the mentioned pancreatic cancer cell lines only, an *in vivo* response is often based on many different cell types and their specific interaction. The formation of desmoplastic reaction by activated pancreatic stellate cells [38–40] and even single components such as cancer associated fibroblasts might contribute to chemoresistance *in vivo*, for instance, via cytoprotective autophagy [41, 42].

Moreover, the following limitations of the study might account for the lacking efficacy of these combination therapies. The used dosage in the present study might be insufficient to achieve therapeutic effects *in vivo*. CHC was applied in other preclinical studies with a dosage of 100–200 mg/kg, daily. These studies observed a significant reduction of tumor progression after CHC treatment in xenografts or syngenic models for breast cancer [43], lung carcinoma [35] and osteosarcoma [44]. Accordingly we did also start with higher dosage of CHC in a preliminary study, however we observed an increased mortality of up to 60% when injecting 60–240 mg/kg CHC on a daily basis [42]. We therefore reduced the CHC dosage to 15 mg/kg. However, this dose might be too low to reduce the tumor growth *in vivo*. The CHC response might also be tumor model specific, since one research group did also not observe a significant reduction of tumor growth, when applying a high dose of 200 mg/kg CHC on a daily basis using a tumor model of triple-negative breast cancer [45].

The anti-cancer efficacy of galloflavin was evaluated on different cell lines *in vitro* [37, 46, 47]. However no anticancer effect of galloflavin alone or in combination with other drugs could be quantified *in vivo* so far [26]. An effective *in vivo* inhibition of LDH activity was observed with a concentration of 80 mg/kg galloflavin in mouse models for acute liver failure [48]. We also used a higher dose of galloflavin (50 mg/kg) at the beginning of our preliminary *in vivo* experiments. However, we observed at these concentrations a precipitation of galloflavin in the peritoneum with an accumulation in the adipose tissue. The finally used concentration of 20 mg/kg galloflavin might be insufficient to achieve anticancer effects *in vivo*.

The used CPI dosage of 25 mg/kg, once per week, provoked a significant reduction of pancreatic tumor progression in xenografts [11, 14] and even a low dosage of 10 mg/kg once a week was effective in a lung carcinoma model [11]. Unfortunately, we could not reproduce these effects when using a comparable dosage of CPI (25 mg/kg once a week). Even by increasing the weekly dose of CPI from 25 mg/kg to 50 mg/kg (5 x 10 mg/kg, Figs 4 and 5), we were not able to see an impairment of tumor growth in the combinatorial treatments. The significant body weight loss of the mice treated with CPI + galloflavin at the end of the experiment (Fig 5H) indicate that a possible increase of dosage or an extension of the treatment period might also impair the health of mice. We therefore abstained to optimize the treatment regime in additional experiments. Besides the applied dosages of the therapeutics, the lack of efficacy of the *in vivo* model, might also be caused by an inefficient intra-tumoral concentration of the drugs.

Another limitation of the study might be the application of an orthotopic mouse model. The analyzed tumor weights had high standard deviations (Figs 3E, 4G and 5G). This heterogeneous tumor progression hinders the quantification of a possible anti-cancer effect. Possibly, efficacy of therapies can be more easily observed in heterotopic animal models (e.g. injecting cancer cells subcutaneously) than in orthotopic models. However, one should be aware that often orthotopic animal models mimic the clinical situation better than heterotopic models [49]. Another limitation might be the low sample size of the analyzed tumors. According to a sample size calculation based on tumor weights of CPI + CHC (mean ± SD: 0.230 g ± 0.190 g) vs. the control group (mean ± SD 0.325 g ± 0.347 g) 108 mice would be necessary to get significant results (assessed Cohen’s d was 0.34). The application of so many animals, for a therapy with questionable anti-cancerous effect might ethically be unjustifiable and disagrees with the 3Rs [50].

The failure of preclinical efficacy of new drugs might also be due to their inappropriate pharmacokinetic properties [51]. These pharmacokinetic and pharmacodynamic aspects might be improved with other drugs [52, 53]. Especially the drug delivery into pancreatic tumors is hindered by the excessive desmoplasia and vascular deficiency [54].

In addition to the present results, the failure to reproduce anticancer efficacy of some drugs *in vivo* is also reported by many other studies [45, 55, 56]. These results highlights the importance of robust *in vivo* testing of anti-cancer drugs.

## Conclusion

The present study analyzed the anti-cancerous efficacy of the TCA-cycle inhibitor CPI in combination with distinct inhibitors of lactate metabolism. Both combinatorial treatments of CPI with either galloflavin, or CHC resulted in a significant impairment of proliferation and a significant induction of cell death in some pancreatic cancer cell lines. However, these anti-cancerous effects of both therapies could not be reproduced *in vivo* in an orthotopic pancreatic cancer mouse model. Even when this study failed to quantify a therapeutic efficacy of both combinatorial treatments *in vivo*, the *in vitro* results suggest that a combined inhibition of these metabolic pathways might still be an innovative approach for cancer therapy. However, inhibitors with better efficacy *in vivo* should be chosen in future studies. CPI can still be used as a potential inhibitor of the TCA-cycle, since the anti-cancerous efficacy was already determined in clinical trials [14, 57]. A combination of CPI with other inhibitors of lactate metabolism, such as the selective MCT-1 inhibitor AZD3965, might be promising [58]. The preclinical safety of AZD3965 was already evaluated and this inhibitor is already used in a preclinical study for solid tumors and different kinds of lymphoma (clinicaltrials.gov: NCT01791595) [59, 60].

## Supporting information

S1 Fig CPI in combination with CHC inhibits the proliferation and induces cell death in a murine and a humane pancreatic cancer cell line. (**A-B**) Quantification of proliferation by BrdU-ELISA, dose response curve after 48 h treatment with CPI-613 (50–400 μM), with and without 5 mM CHC (dotted line indicates mean value of absorbance for control treated cells with DMSO only) for the humane pancreatic cancer cell line Mia Paca and the murine cell line Panc02. (**C-D**) Analysis of proliferation in Mia Paca, or Panc02 cells, after treatment for 48 h with the respective vehicle DMSO (C), 200 μM CPI (CPI), CPI and CHC (CPI+CHC) or 5 mM CHC (CHC). (**E**) Cell death was quantified by trypan blue assay for Mia Paca cells, when treating the cells with the vehicle DMSO, 300 μM CPI, CPI in combination with CHC, or CHC. (**F**) For Panc02 cells the cell death was quantified by treatment of 250 μM CPI and 5 mM CHC. Statistics were performed, either by ordinary One Way ANOVA (C-D), or Kruskal Wallis Test (E-F), p -F), p ≤ 0.05 was considered to be significant. A-D: n = 3, E: n = 10–11, F: n = 7.
(TIF)

S2 Fig CPI in combination with galloflavin exhibit anticancerouse effects on a humane and a murine pancreatic cancer cell line. (**A-B**) Analysis of proliferation by BrdU-ELISA after treatment for 24 h with CPI-613 (100–400 μM) only, as well as in combination with 50 μM galloflavin for the humane Mia Paca cell line, or with 30 μM galloflavin for the murine Pan02 cells (dotted line indicates mean value of absorbance for control treated cells with DMSO). (**C-D**) Analysis of proliferation respectively for Mia Paca, or Panc02 cells, after treatment for 24 h with the respective vehicle DMSO (C), 200 μM CPI (CPI), CPI and galloflavin (CPI+GAL) or galloflavin (GAL) only. (**E**) Cell death was quantified by trypan blue assay for Mia Paca cells, when treating the cells with the vehicle DMSO, 250 μM CPI in combination with 30 μM galloflavin. (**F**) For Panc02 cells the cell death was quantified by treatment of 225 μM CPI and 50 μM galloflavin (F). Statistics were performed either by ordinary One Way ANOVA (C-E), or Kruskal Wallis Test (F), p ≤ 0.05 was considered to be significant. A, C: n = 6, B, D-F: n = 8.
(TIF)

S3 Fig The metabolic targets PDH and α-KGDH are expressed in 6606PDA cells. (**A**) The expression of PDH was assessed by western blot in 6606PDA cells after treatment with medium only (Med), the vehicle DMSO (C) or 300 μM CPI for 3 h. (**B**) The enzymatic activity of a-KGDH was analyzed by a-KGDH colorimetric assay kit, after treating the 6606PDA cells for 4 h with 300 μM CPI or the respective concentration of the vehicle DMSO (C). Each sample was calculated as percentage to control. A: n = 2 independent western blots were performed, B: n = 6 independent replicates of treated cells at different passages were quantified by one colorimetric assay.
(TIF)

S4 Fig Immunohistochemistry detects the metabolic targets of CPI, galloflavin and CHC in tumor tissue after sham treatment, therapeutic intervention and the respective conjugate control for each staining. (**A-F**) Immunostaining of the metabolic targets of CPI, pyruvate dehydrogenase (PDH) and alpha-Ketoglutarate dehydrogenase (α-KGDH), either in tumors after sham treatment, CPI+CHC intervention, CPI+GAL treatment or the conjugate controls for each staining. (**G-I**) Distribution of the enzyme lactate dehydrogenase (LDH) in murine tumors after sham treatment, CPI+GAL and the conjugate control. (**J-L**) Detection of monocarboxylate transporter (MCT-4), as metabolic target of CHC, either in tumors of sham treated mice, after CPI+CHC treatment or the conjugate control. The scale bar represents 50 μm.
(TIF)

S1 Raw images Uncropped images of all western blot gels. (PDF)

S1 Data Raw data of all measured parameters. (XLSX)
