# Supplementary figures and images for: Targeting pancreatic cancer with combinatorial treatment of CPI-613 and inhibitors of lactate metabolism
Source: PLoS One. 2022 Apr 22;17(4):e0266601. doi: 10.1371/journal.pone.0266601 (PMC9032382; doi:10.1371/journal.pone.0266601)

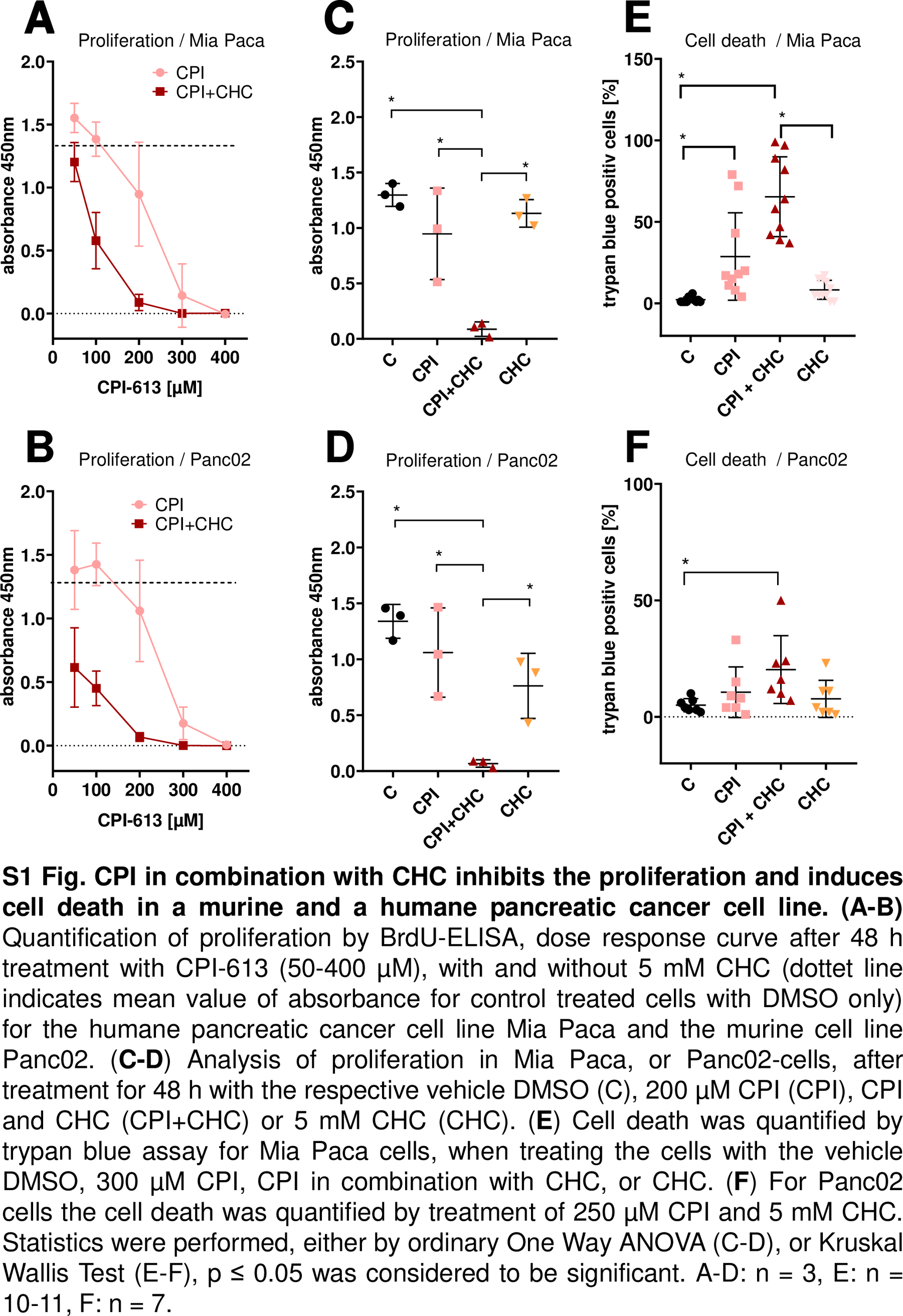

Supplement: S1 Fig — (A-B) Quantification of proliferation by BrdU-ELISA, dose response curve after 48 h treatment with CPI-613 (50–400 μM), with and without 5 mM CHC (dotted line indicates mean value of absorbance for control treated cells with DMSO only) for the humane pancreatic cancer cell line Mia Paca and the murine cell line Panc02. (C-D) Analysis of proliferation in Mia Paca, or Panc02 cells, after treatment for 48 h with the respective vehicle DMSO (C), 200 μM CPI (CPI), CPI and CHC (CPI+CHC) or 5 mM CHC (CHC). (E) Cell death was quantified by trypan blue assay for Mia Paca cells, when treating the cells with the vehicle DMSO, 300 μM CPI, CPI in combination with CHC, or CHC. (F) For Panc02 cells the cell death was quantified by treatment of 250 μM CPI and 5 mM CHC. Statistics were performed, either by ordinary One Way ANOVA (C-D), or Kruskal Wallis Test (E-F), p -F), p ≤ 0.05 was considered to be significant. A-D: n = 3, E: n = 10–11, F: n = 7. (TIF) [file pone.0266601.s001.tif]

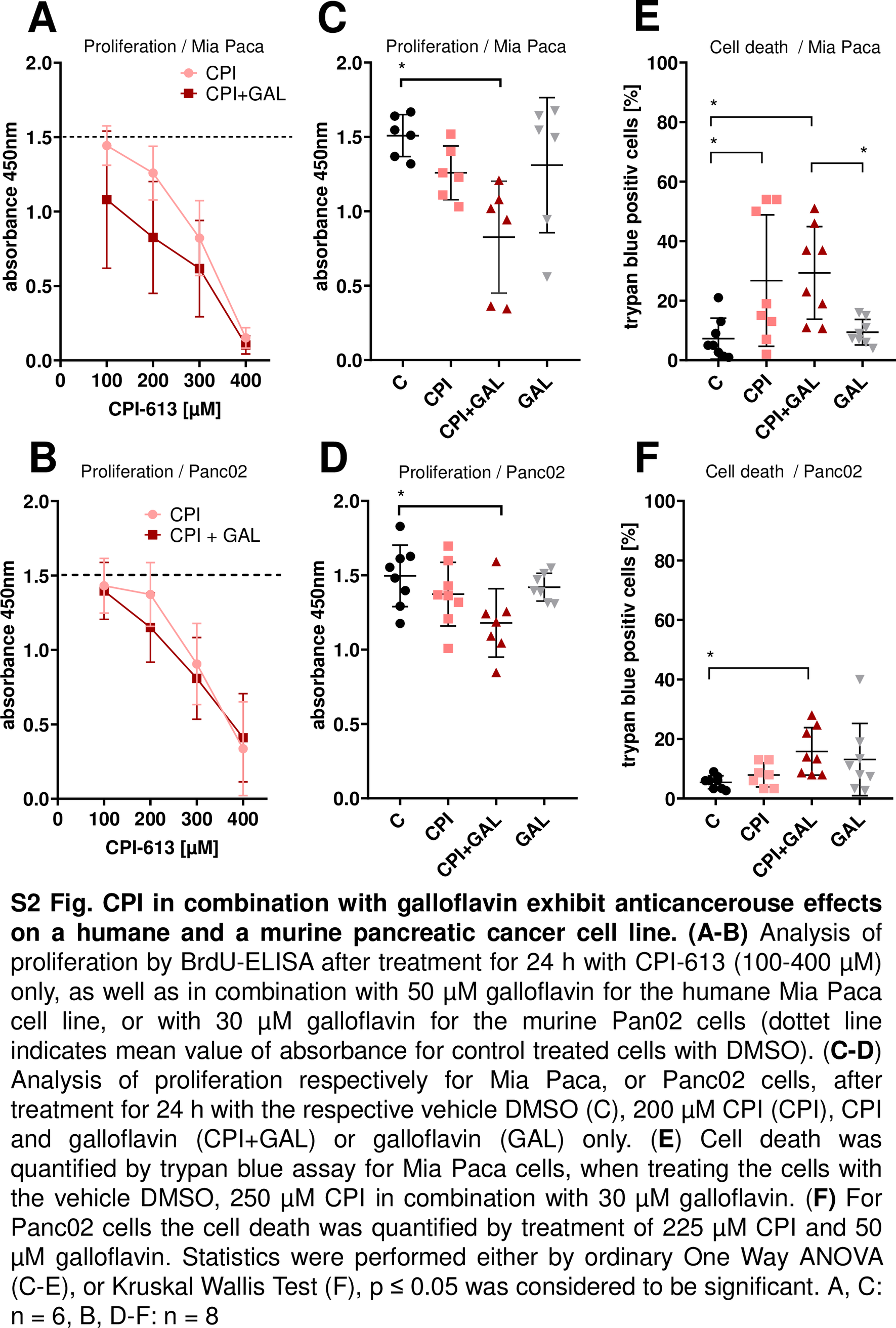

Supplement: S2 Fig — (A-B) Analysis of proliferation by BrdU-ELISA after treatment for 24 h with CPI-613 (100–400 μM) only, as well as in combination with 50 μM galloflavin for the humane Mia Paca cell line, or with 30 μM galloflavin for the murine Pan02 cells (dotted line indicates mean value of absorbance for control treated cells with DMSO). (C-D) Analysis of proliferation respectively for Mia Paca, or Panc02 cells, after treatment for 24 h with the respective vehicle DMSO (C), 200 μM CPI (CPI), CPI and galloflavin (CPI+GAL) or galloflavin (GAL) only. (E) Cell death was quantified by trypan blue assay for Mia Paca cells, when treating the cells with the vehicle DMSO, 250 μM CPI in combination with 30 μM galloflavin. (F) For Panc02 cells the cell death was quantified by treatment of 225 μM CPI and 50 μM galloflavin (F). Statistics were performed either by ordinary One Way ANOVA (C-E), or Kruskal Wallis Test (F), p ≤ 0.05 was considered to be significant. A, C: n = 6, B, D-F: n = 8. (TIF) [file pone.0266601.s002.tif]

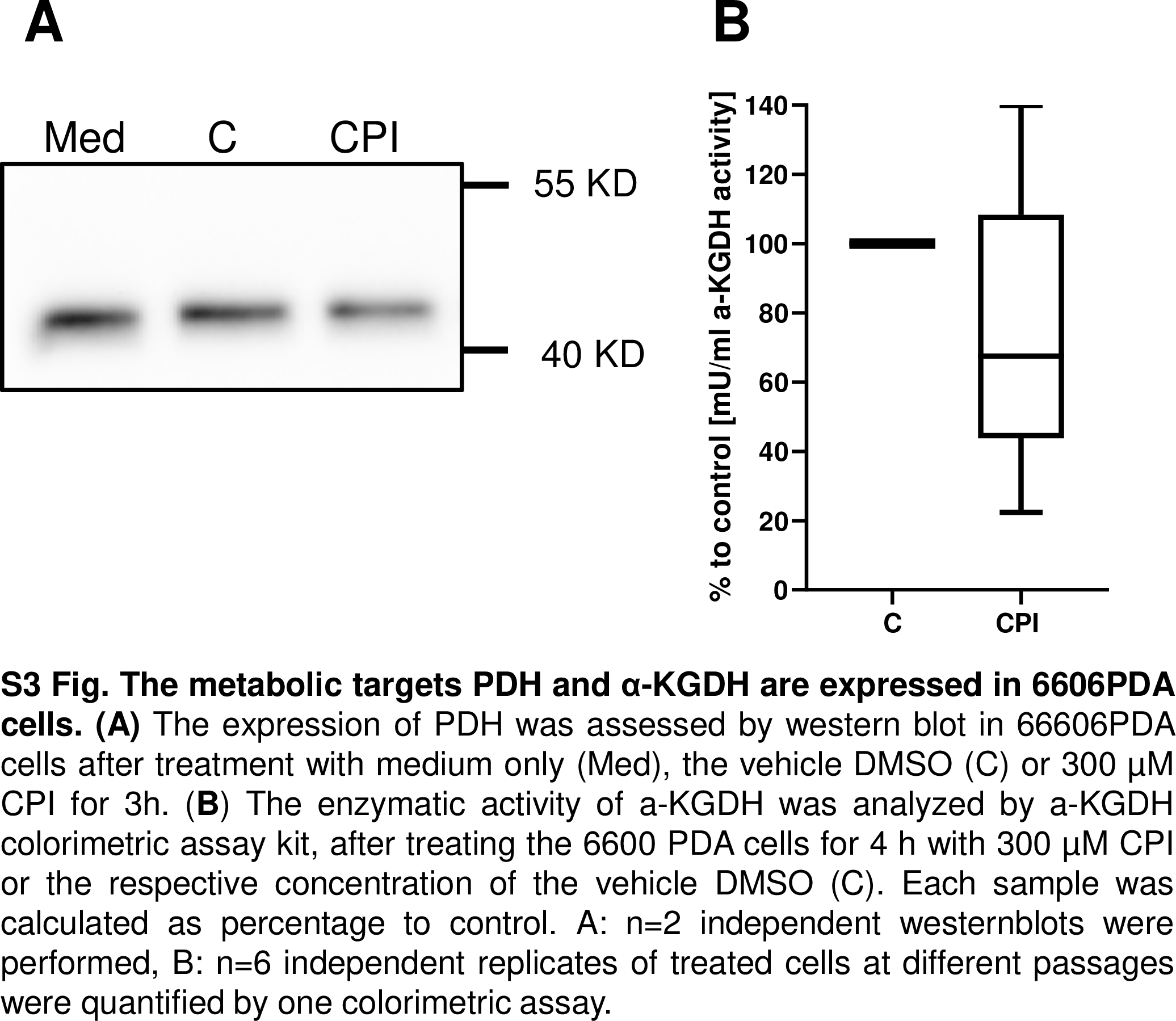

Supplement: S3 Fig — (A) The expression of PDH was assessed by western blot in 6606PDA cells after treatment with medium only (Med), the vehicle DMSO (C) or 300 μM CPI for 3 h. (B) The enzymatic activity of a-KGDH was analyzed by a-KGDH colorimetric assay kit, after treating the 6606PDA cells for 4 h with 300 μM CPI or the respective concentration of the vehicle DMSO (C). Each sample was calculated as percentage to control. A: n = 2 independent western blots were performed, B: n = 6 independent replicates of treated cells at different passages were quantified by one colorimetric assay. (TIF) [file pone.0266601.s003.tif]

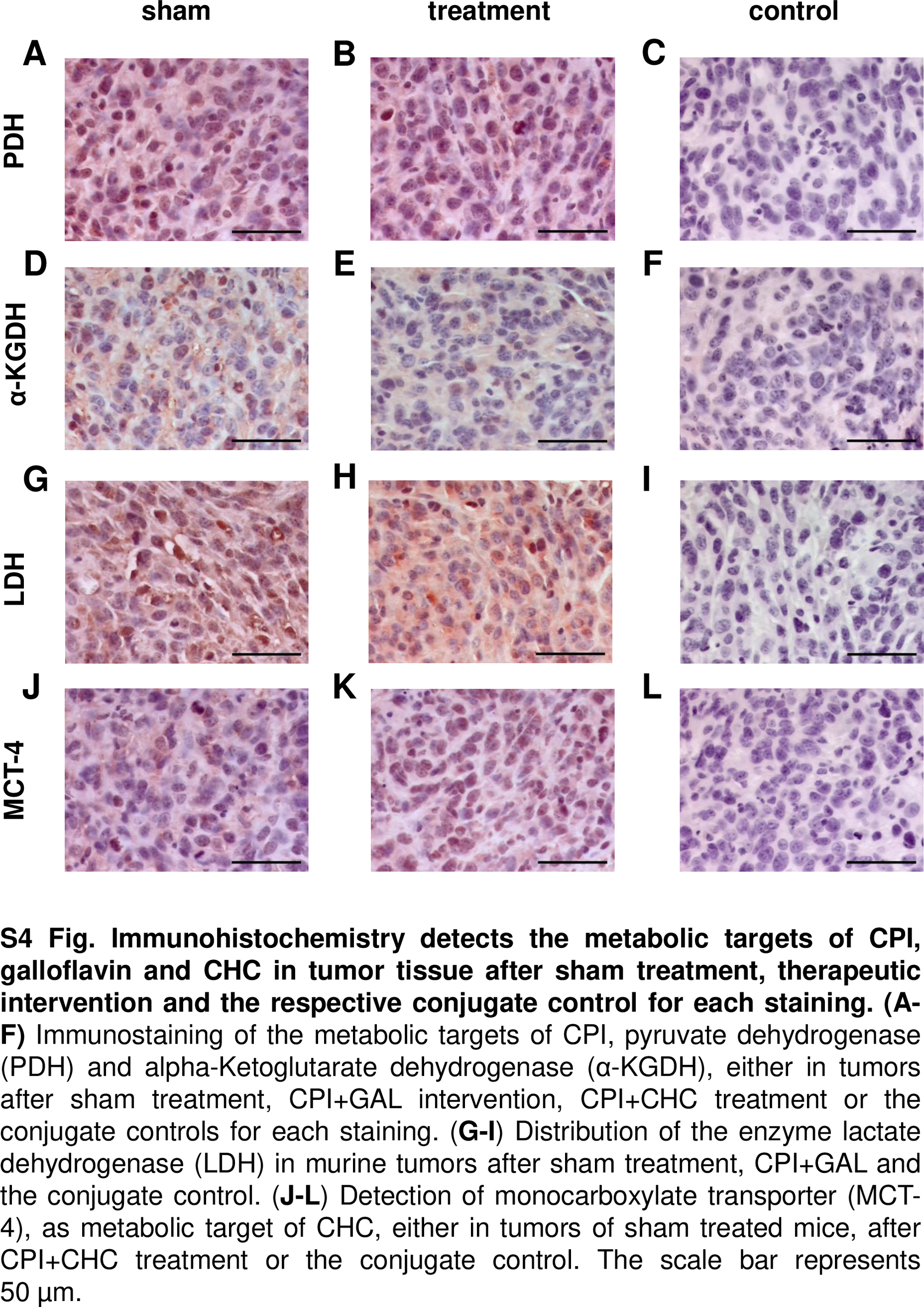

Supplement: S4 Fig — (A-F) Immunostaining of the metabolic targets of CPI, pyruvate dehydrogenase (PDH) and alpha-Ketoglutarate dehydrogenase (α-KGDH), either in tumors after sham treatment, CPI+CHC intervention, CPI+GAL treatment or the conjugate controls for each staining. (G-I) Distribution of the enzyme lactate dehydrogenase (LDH) in murine tumors after sham treatment, CPI+GAL and the conjugate control. (J-L) Detection of monocarboxylate transporter (MCT-4), as metabolic target of CHC, either in tumors of sham treated mice, after CPI+CHC treatment or the conjugate control. The scale bar represents 50 μm. (TIF) [file pone.0266601.s004.tif]
