# Supplementary material for: Targeting pancreatic cancer with combinatorial treatment of CPI-613 and inhibitors of lactate metabolism
Source: PLoS One. 2022 Apr 22;17(4):e0266601. doi: 10.1371/journal.pone.0266601 (PMC9032382; doi:10.1371/journal.pone.0266601)
Supplement: S1 Raw images — (PDF) [file pone.0266601.s005.pdf]

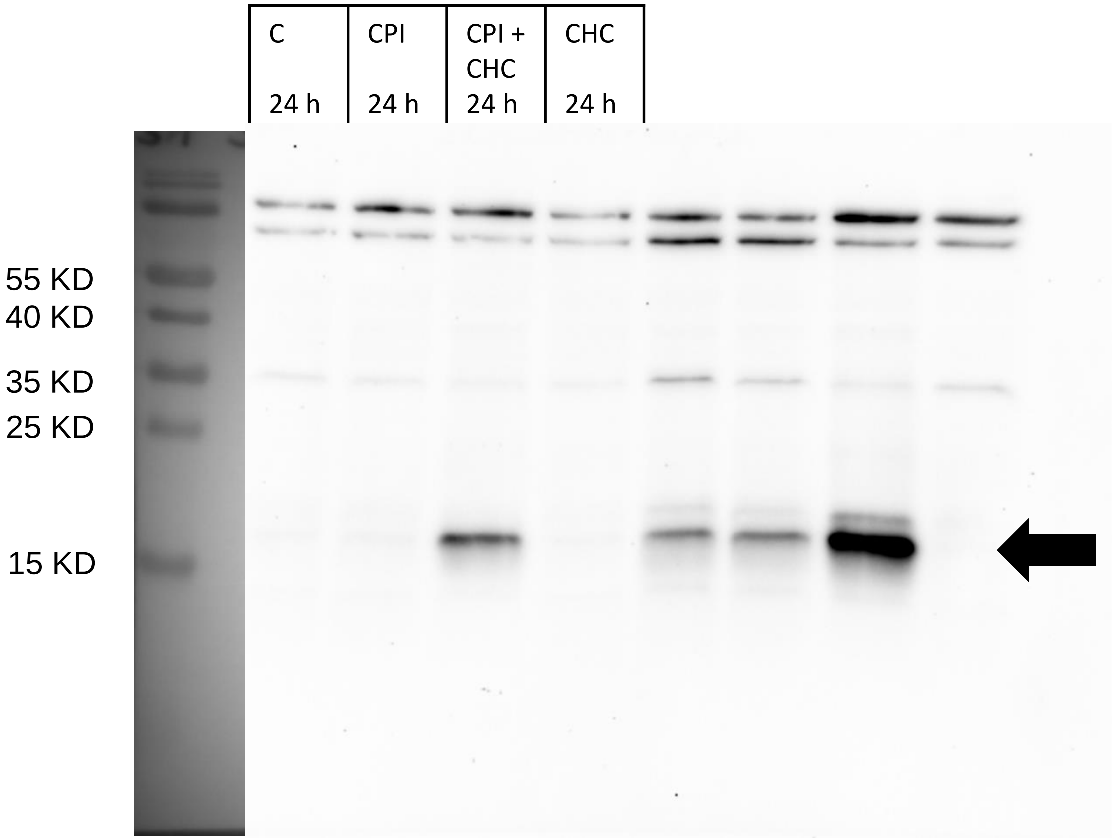

Cleaved caspase 3 17-19 KD: 12.12.17, 35 µg Protein, 14% acrylamid gel, #9661 Cell Signaling Technology (1:1000), #7024 Cell Signaling Technology (1:20:000) , 500.000 seconds exposure time (Fig 1D).

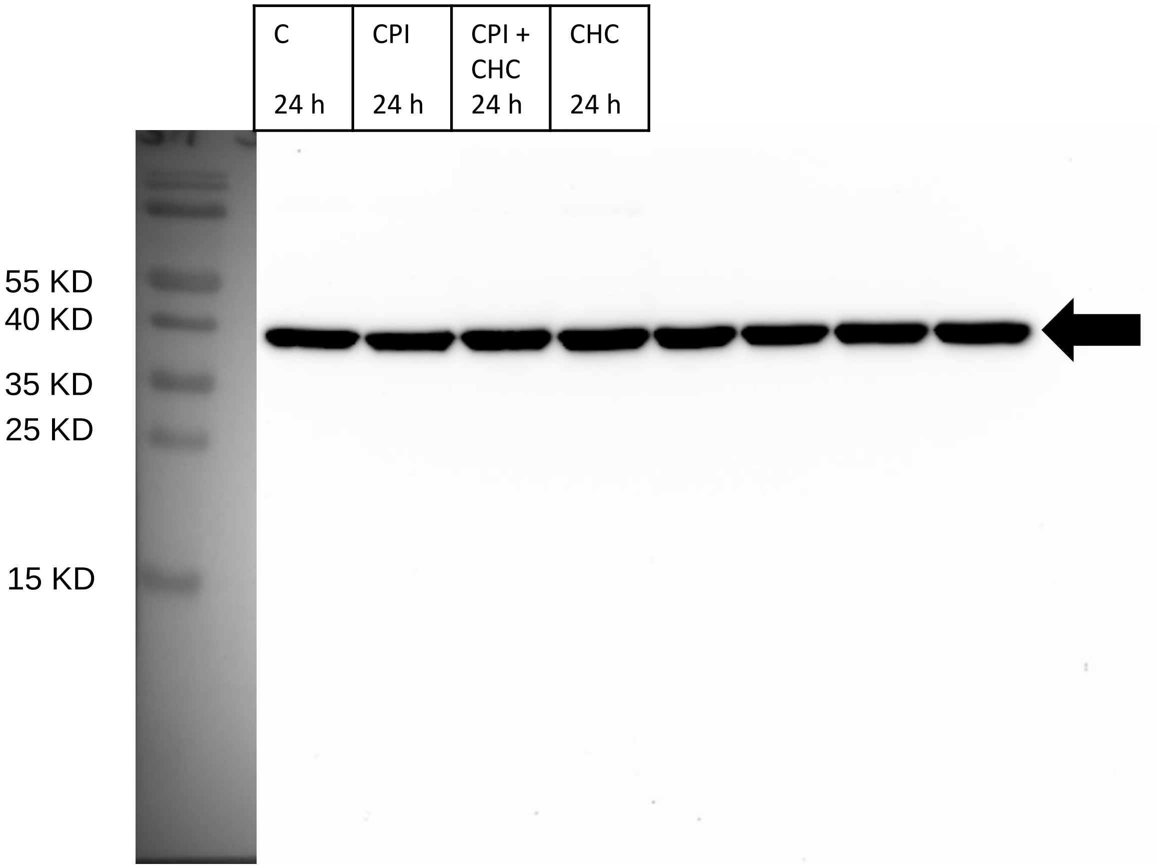

β-actin 42 KD: 13.12.17, 40 µg Protein, 14% acrylamid gel, A5441 Sigma-Aldrich, (1:20.000), 9044 Sigma-Aldrich (1:60:000), 100.000 seconds exposure time (Fig 1D).

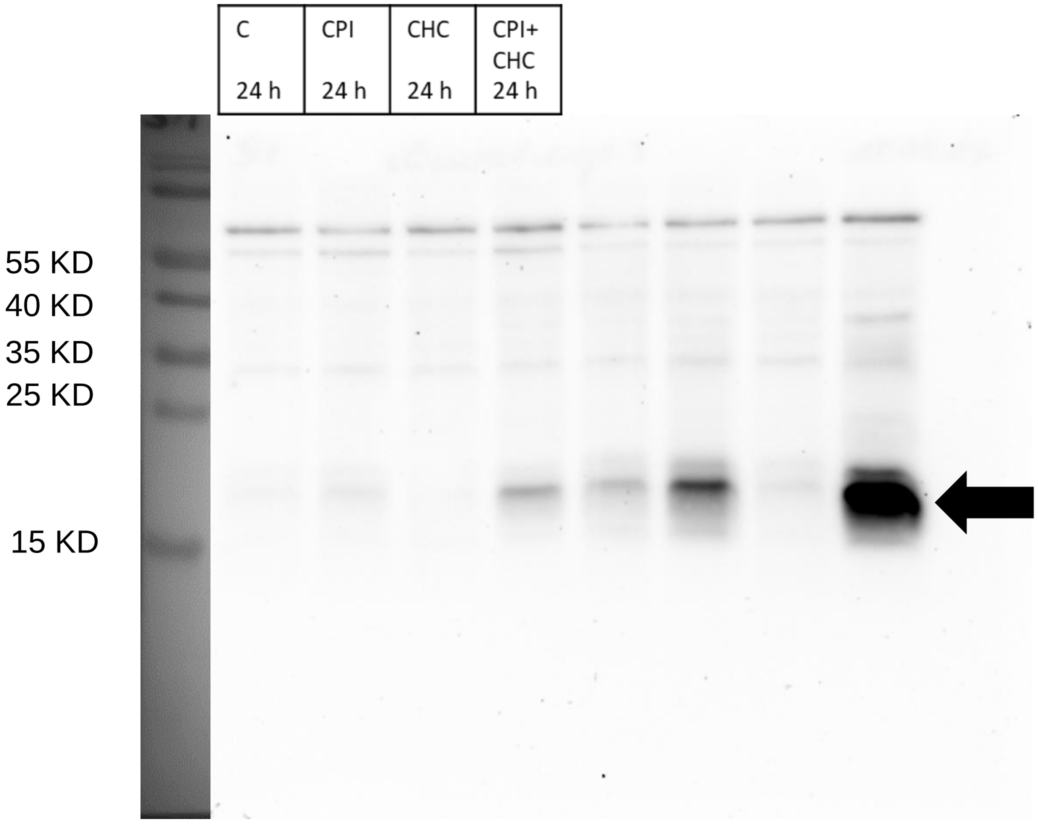

Cleaved caspase 3 17-19 KD: 16.11.17, 35 µg Protein, 14% acrylamid gel, #9661 Cell Signaling Technology (1:1000), #7024 Cell Signaling Technology (1:20:000) , 500.000 seconds exposure time.

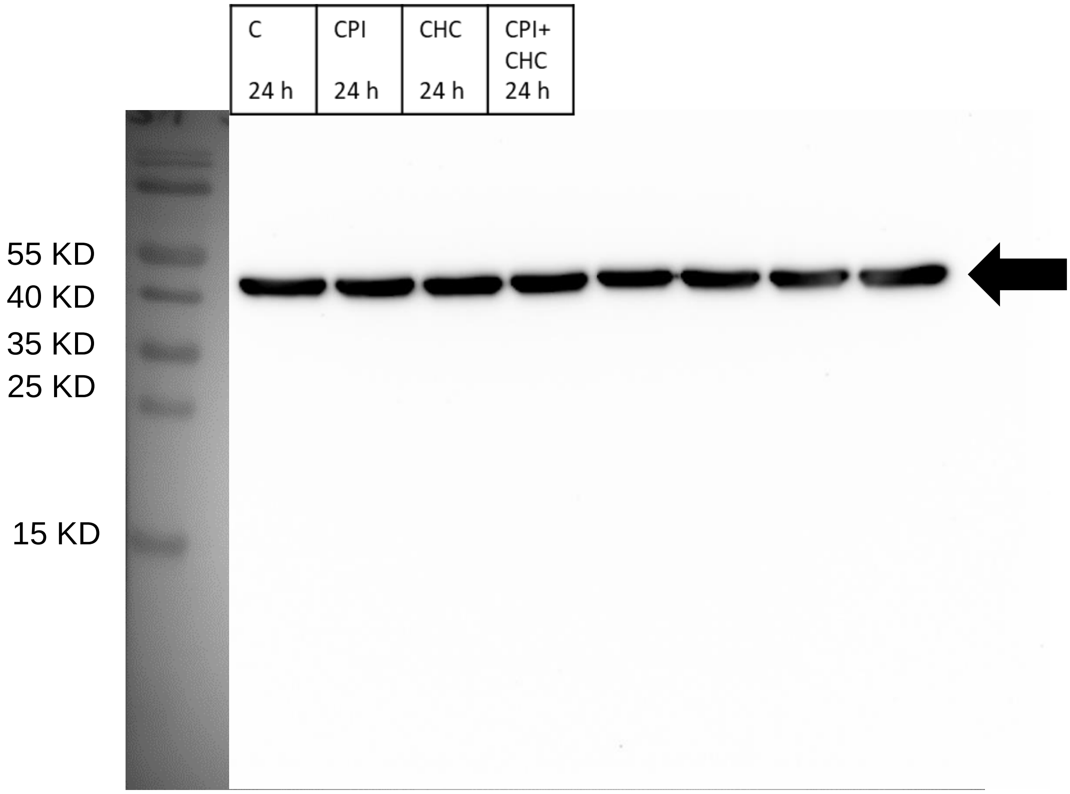

β-actin 42 KD: 20.12.17, 40 µg Protein, 14% acrylamid gel, A5441 Sigma-Aldrich, (1:20.000), 9044 Sigma-Aldrich (1:60:000), 100.000 seconds exposure time.

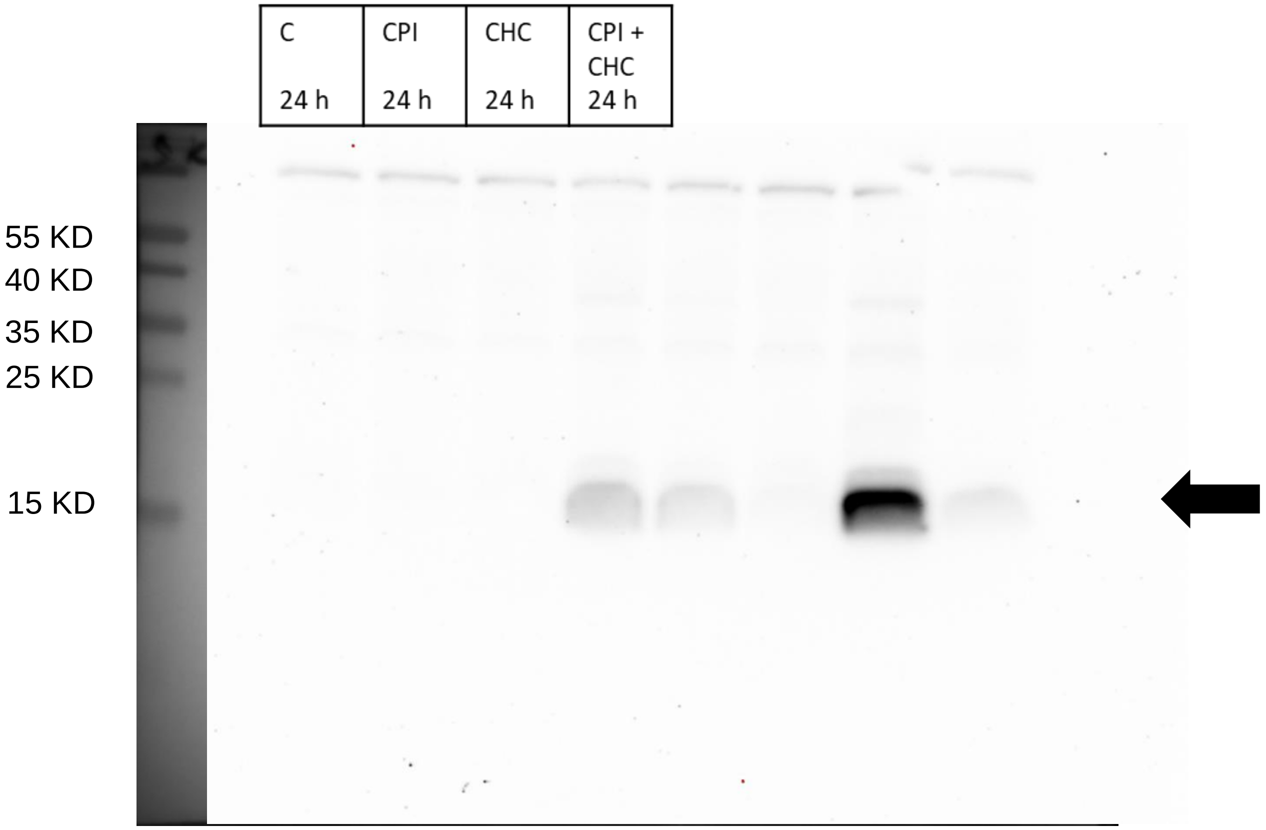

Cleaved caspase 3 17-19 KD: 12.10.17, 40 µg Protein, 14% acrylamid gel, #9661 Cell Signaling Technology (1:1000), #7024 Cell Signaling Technology (1:20:000) , 500.000 seconds exposure time.

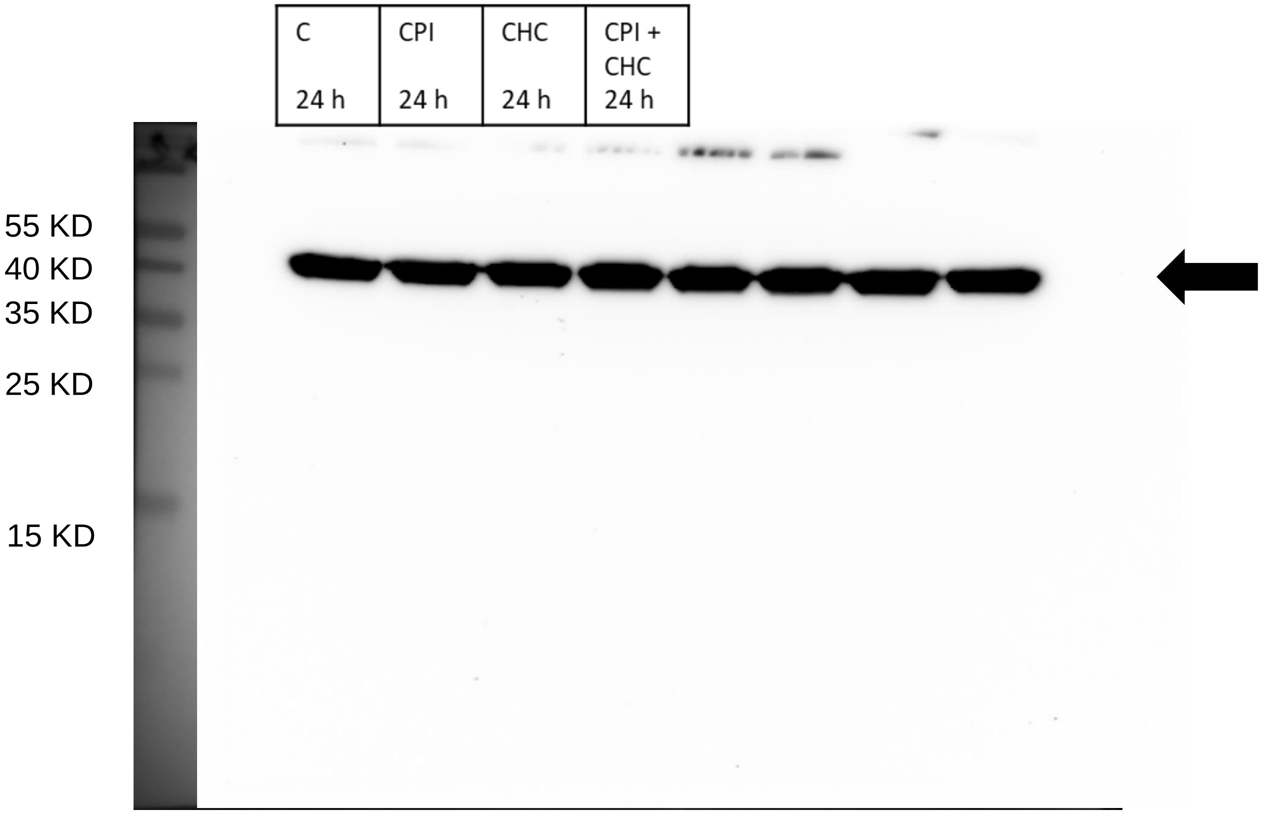

β-actin 42 KD: 13.10.17, 40 µg Protein, 14% acrylamid gel, A5441 Sigma-Aldrich, (1:20.000), 9044 Sigma-Aldrich (1:60:000), 65.000 seconds exposure time.

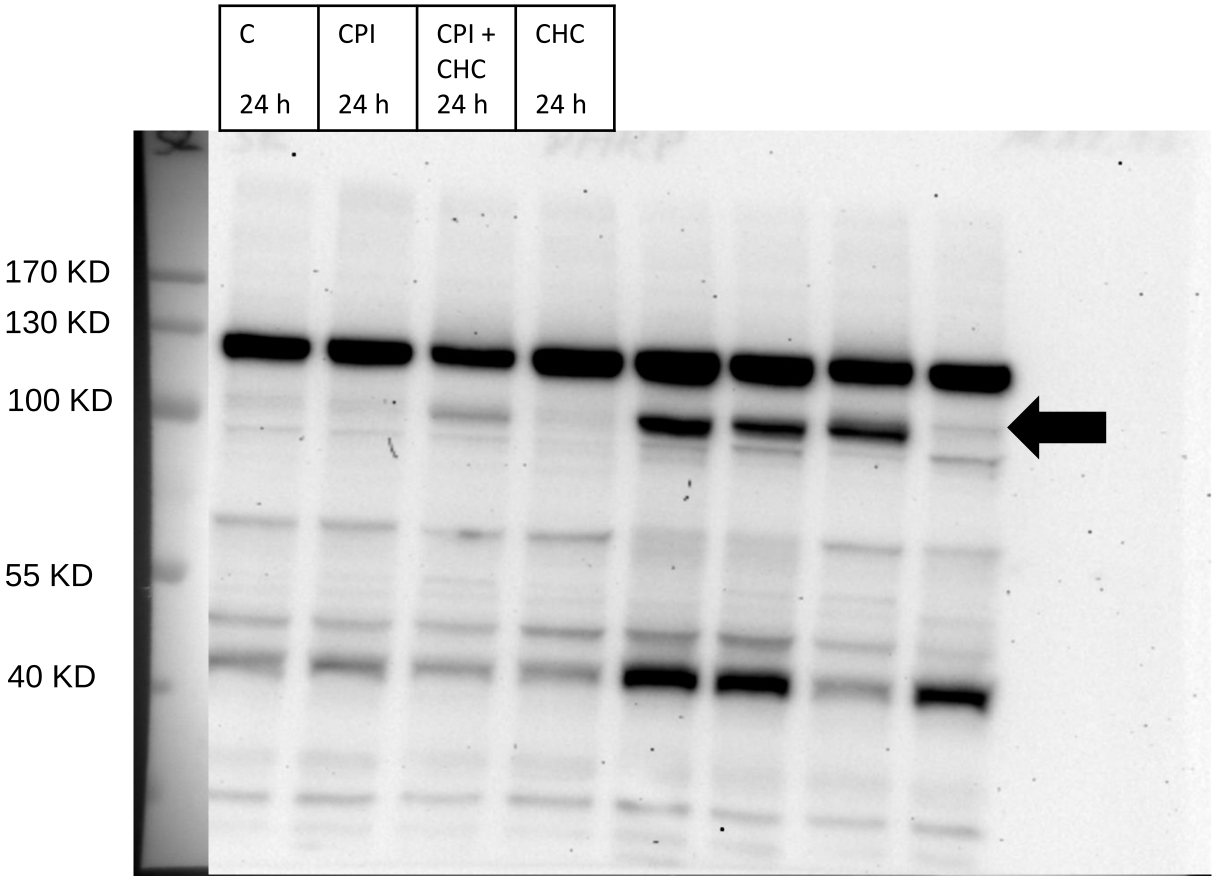

Cleaved parp 116 KD: 12.12.17, 40 µg Protein, 8% acrylamid gel, #9525 Cell Signaling Technology (1:1000), #7024 Cell Signaling Technology (1:20:000) , 200.000 seconds exposure time (Fig 1 E).

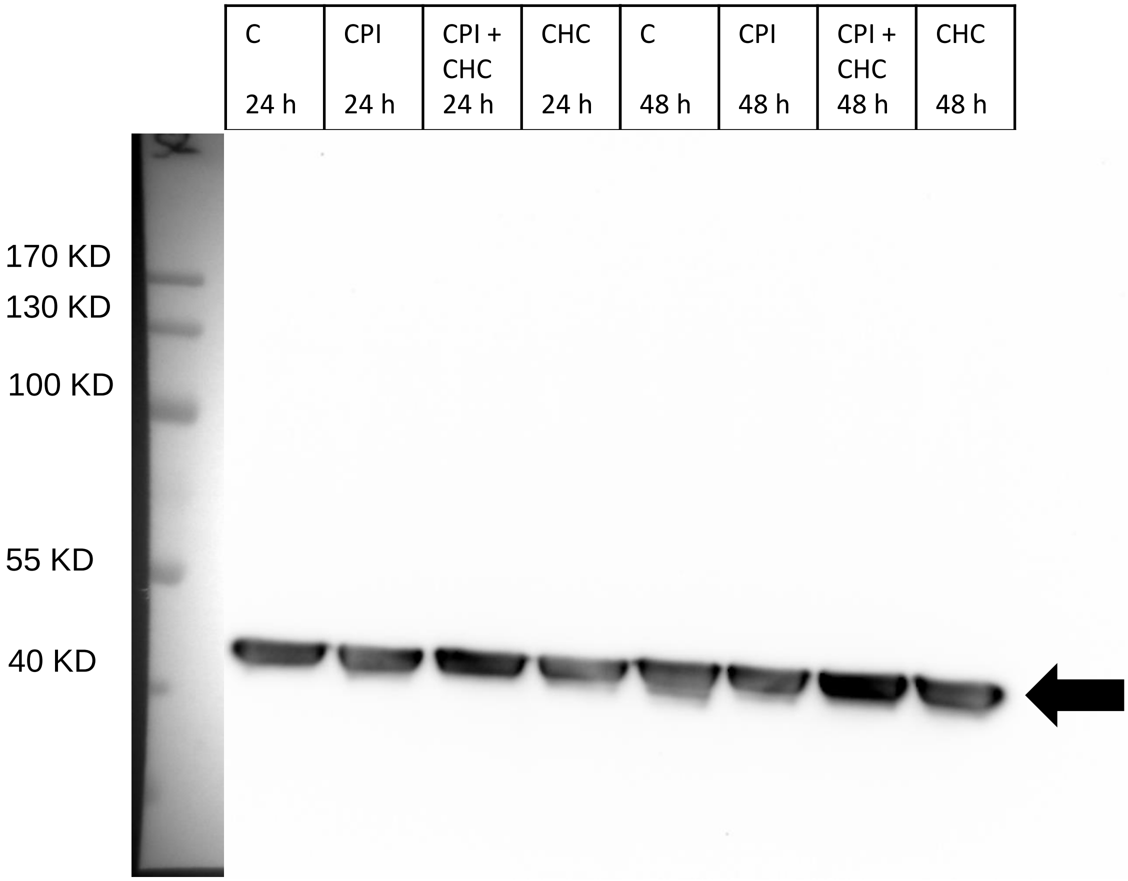

β-actin 42 KD: 13.12.17, 40 µg Protein, 8% acrylamid gel, A5441 Sigma-Aldrich, (1:20.000), 9044 Sigma-Aldrich (1:60:000), 100.000 seconds exposure time. (Fig 1 E)

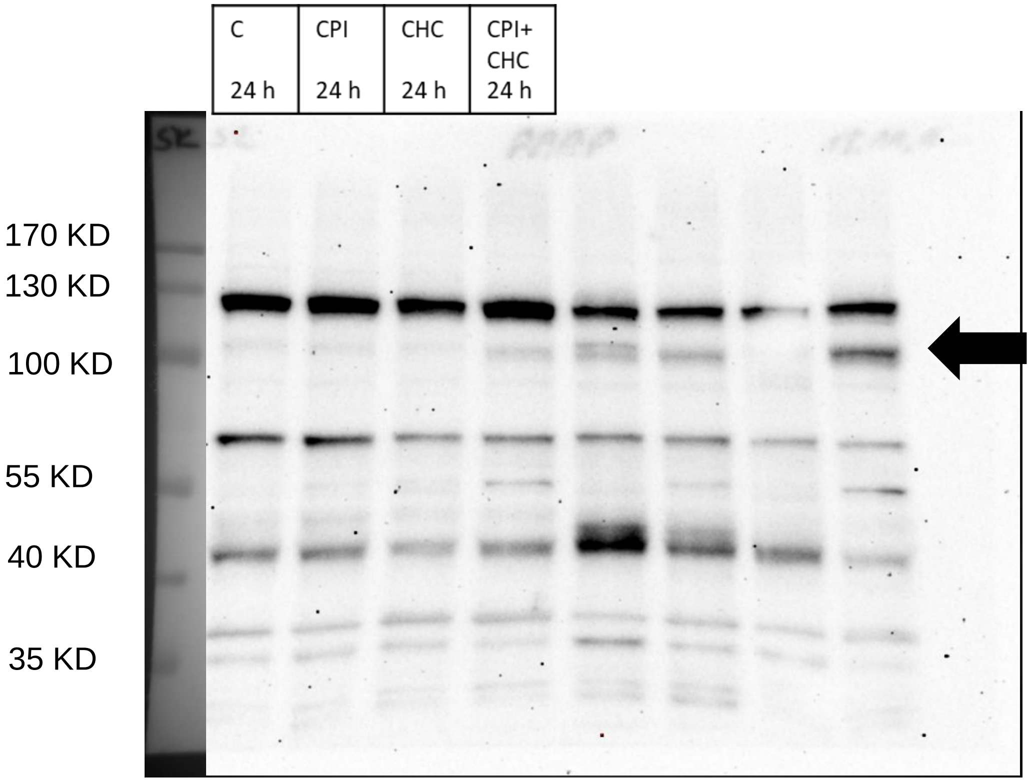

Cleaved parp 116 KD: 16.11.17, 40 µg Protein, 8% acrylamid gel, #9525 Cell Signaling Technology (1:1000), #7024 Cell Signaling Technology (1:20:000) , 500.000 seconds exposure time.

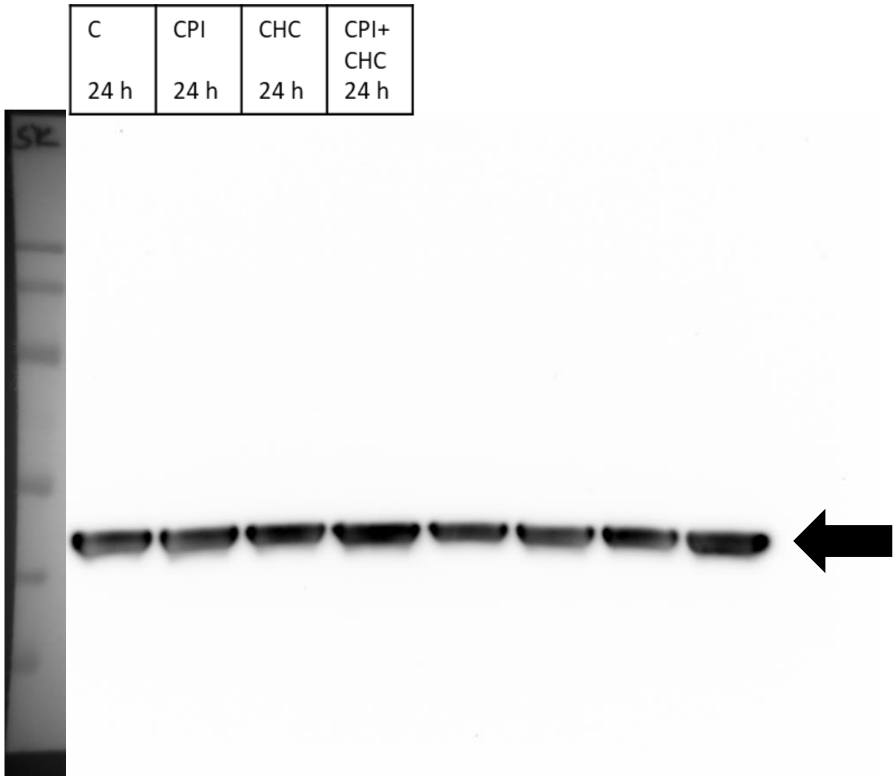

β-actin 42 KD: 20.11.17, 40 µg Protein, 8% acrylamid gel, A5441 Sigma-Aldrich, (1:20.000), 9044 Sigma-Aldrich (1:60:000), 100.000 seconds exposure time.

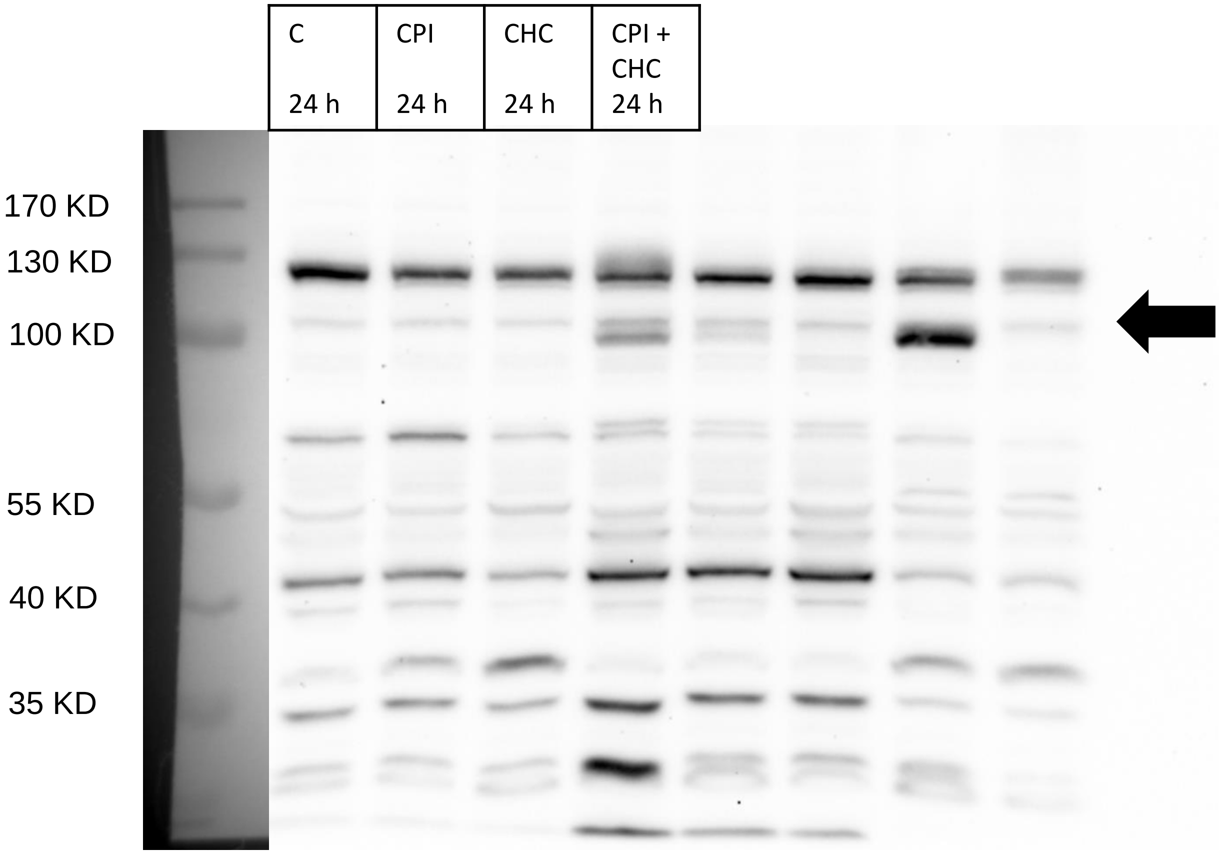

Cleaved parp 116 KD: 12.10.17, 40 µg Protein, 8% acrylamid gel, #9525 Cell Signaling Technology (1:1000), #7024 Cell Signaling Technology (1:20:000) , 200.000 seconds exposure time.

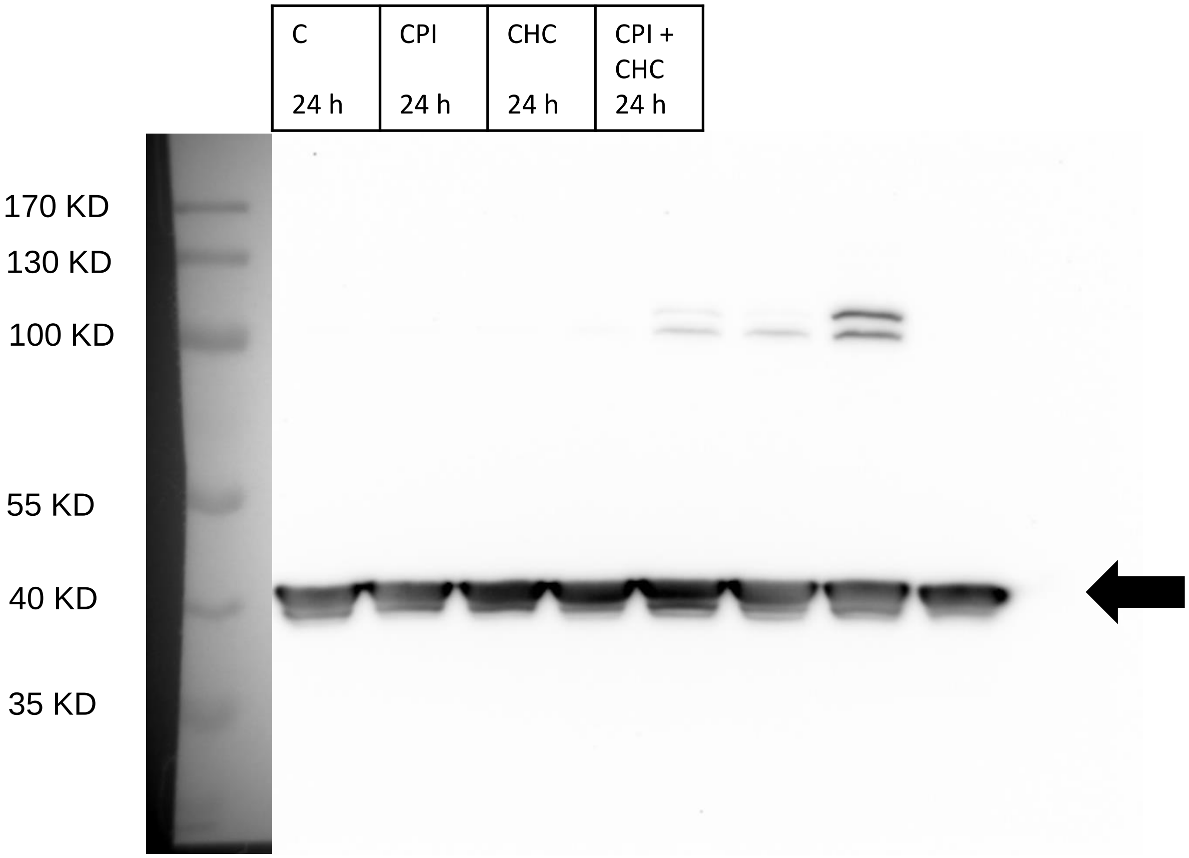

β-actin 42 KD: 13.10.17, 40 µg Protein, 8% acrylamid gel, A5441 Sigma-Aldrich, (1:20.000), 9044 Sigma-Aldrich (1:60:000), 65.000 seconds exposure time.

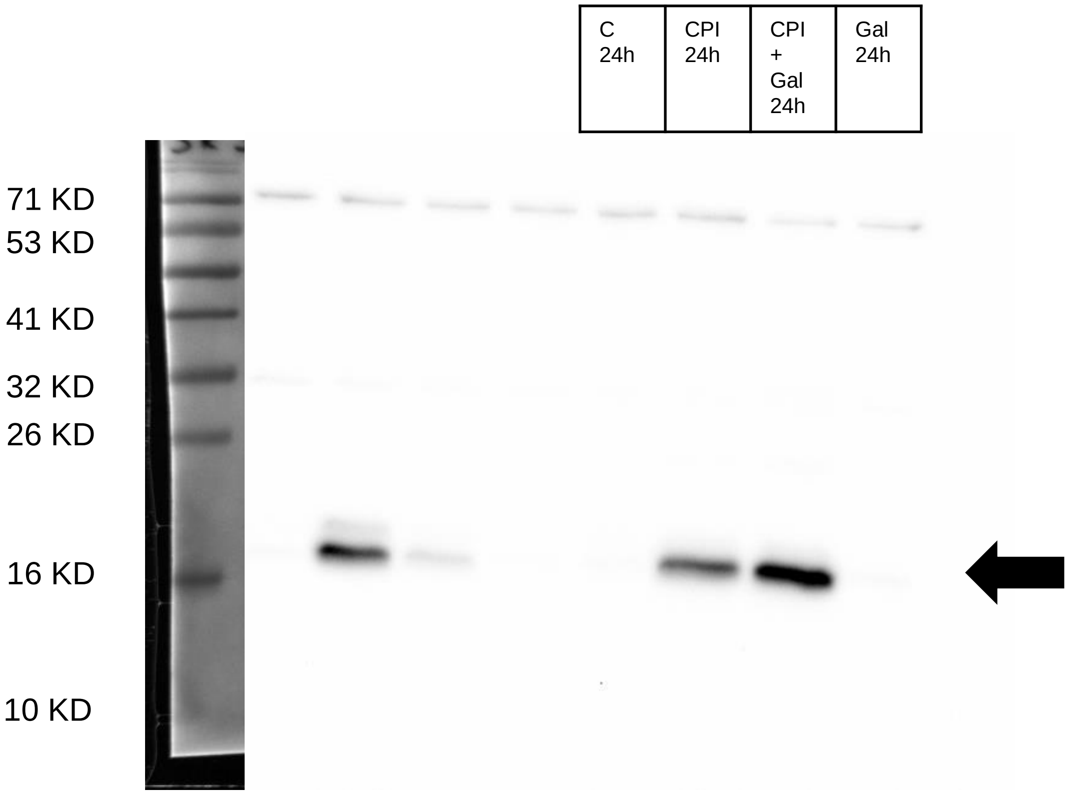

Cleaved caspase 3 17-19 KD: 21.01.20, 25 µg Protein, 14% acrylamid gel, #9661 Cell Signaling Technology (1:1000), #7024 Cell Signaling Technology (1:20:000) , 25.000 seconds exposure time (Fig. 2E).

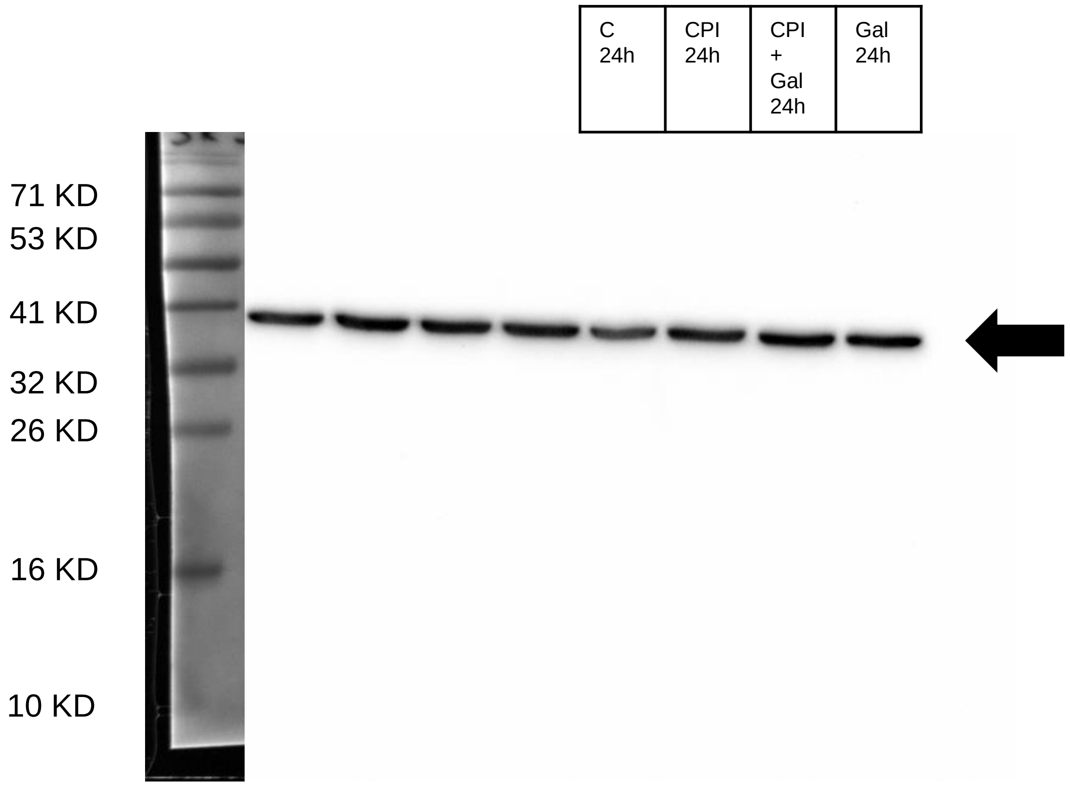

β-actin 42 KD: 22.01.20, 25 µg Protein, 14% acrylamid gel, A5441 Sigma-Aldrich, 1:20.000), 9044 Sigma-Aldrich (1:60:000), 25.000 seconds exposure time (Fig 2 E).

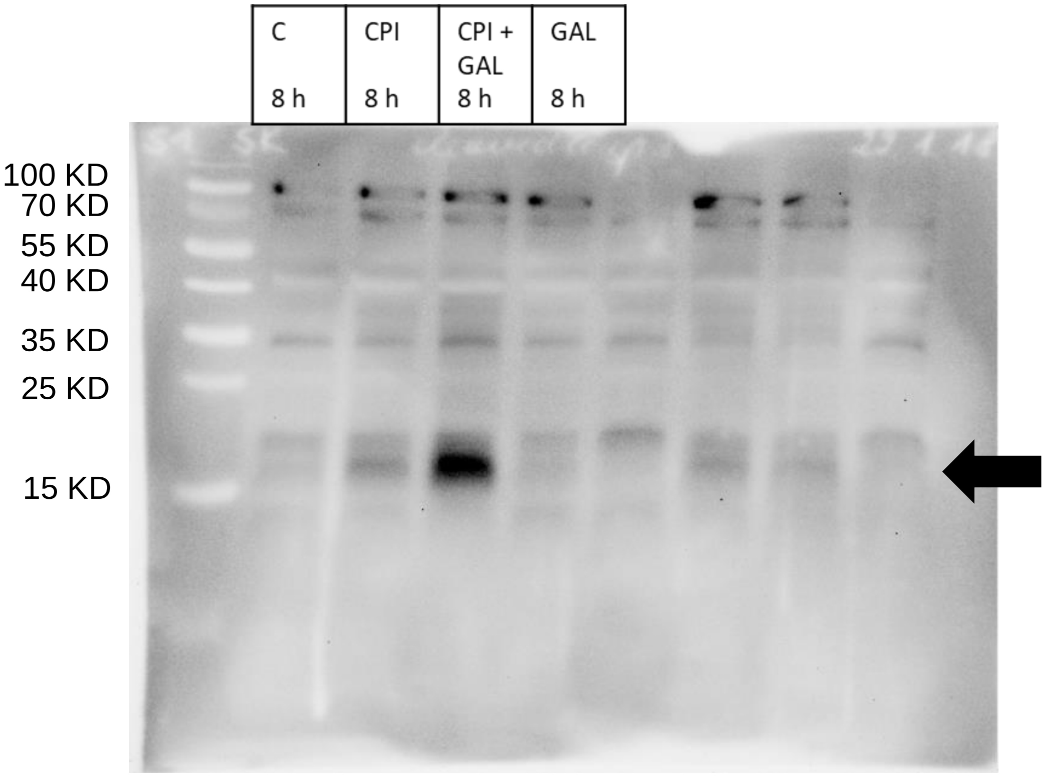

Cleaved caspase 3 17-19 KD: 30.01.18, 40 µg Protein, 14% acrylamid gel, #9661 Cell Signaling Technology (1:1000), #7024 Cell Signaling Technology (1:20:000) , 500.000 seconds exposure time (**Fig 2D**).

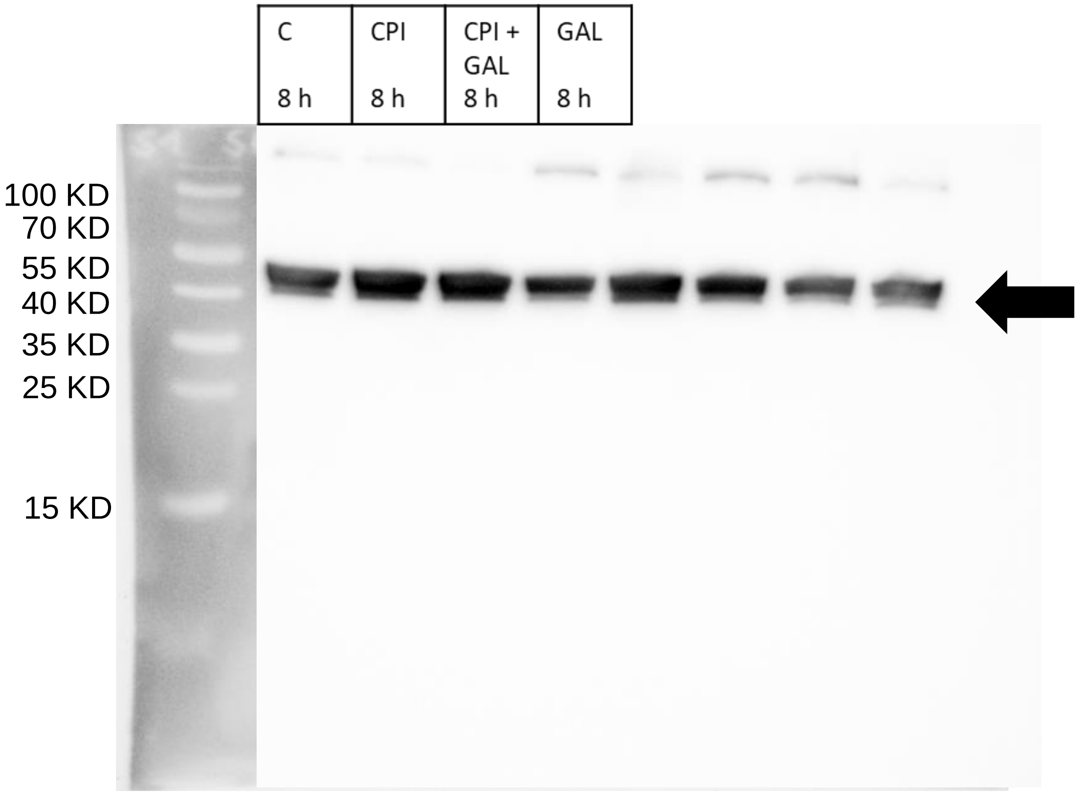

β-actin 42 KD: 01.02.18, 40 µg Protein, 14% acrylamid gel, A5441 Sigma-Aldrich, (1:20.000), 9044 Sigma-Aldrich (1:60:000), 100.000 seconds exposure time (**Fig 2D**).

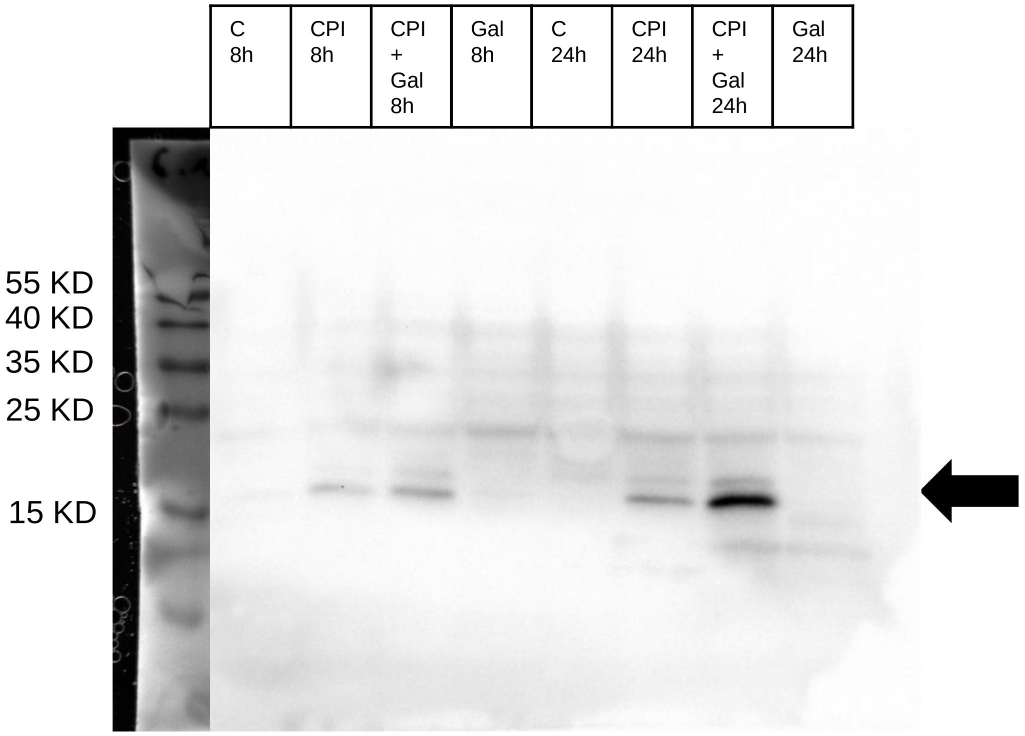

Cleaved caspase 3 17-19 KD: 07.10.21, 40 µg Protein, 14% acrylamid gel, #9661 Cell Signaling Technology (1:1000), #7024 Cell Signaling Technology (1:20:000) , 500.000 seconds exposure time.

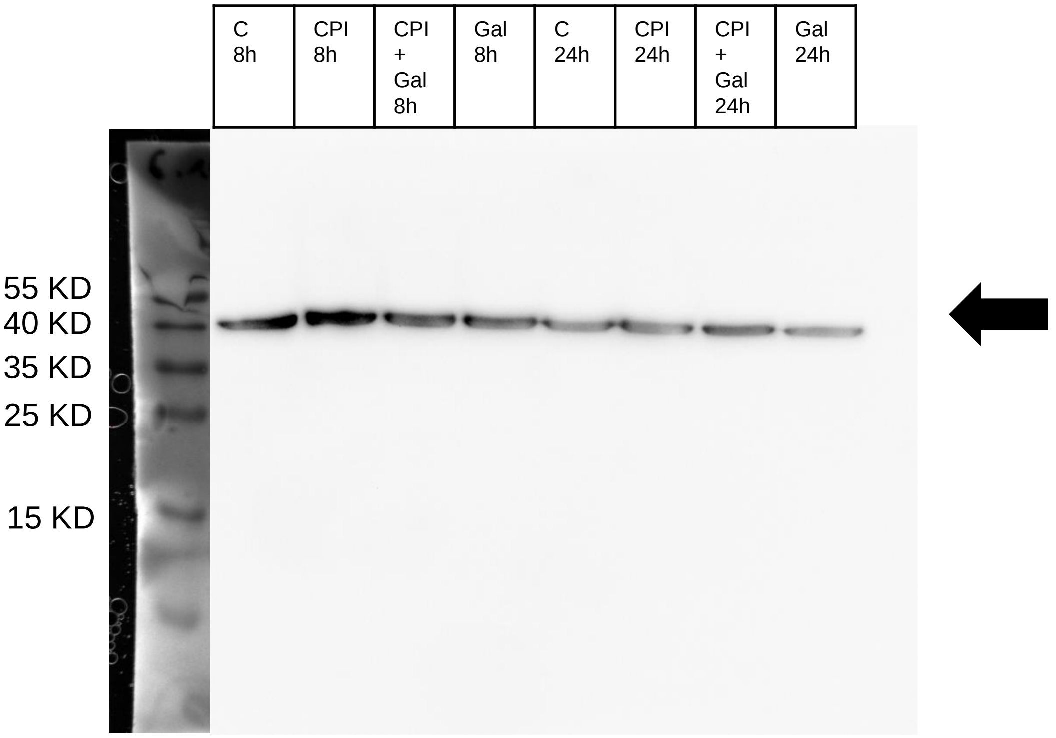

β-actin 42 KD: 15.10.21, 40 µg Protein, 14% acrylamid gel, A5441 Sigma-Aldrich, 1:20.000), 9044 Sigma-Aldrich (1:60:000), 65.000 seconds exposure time.

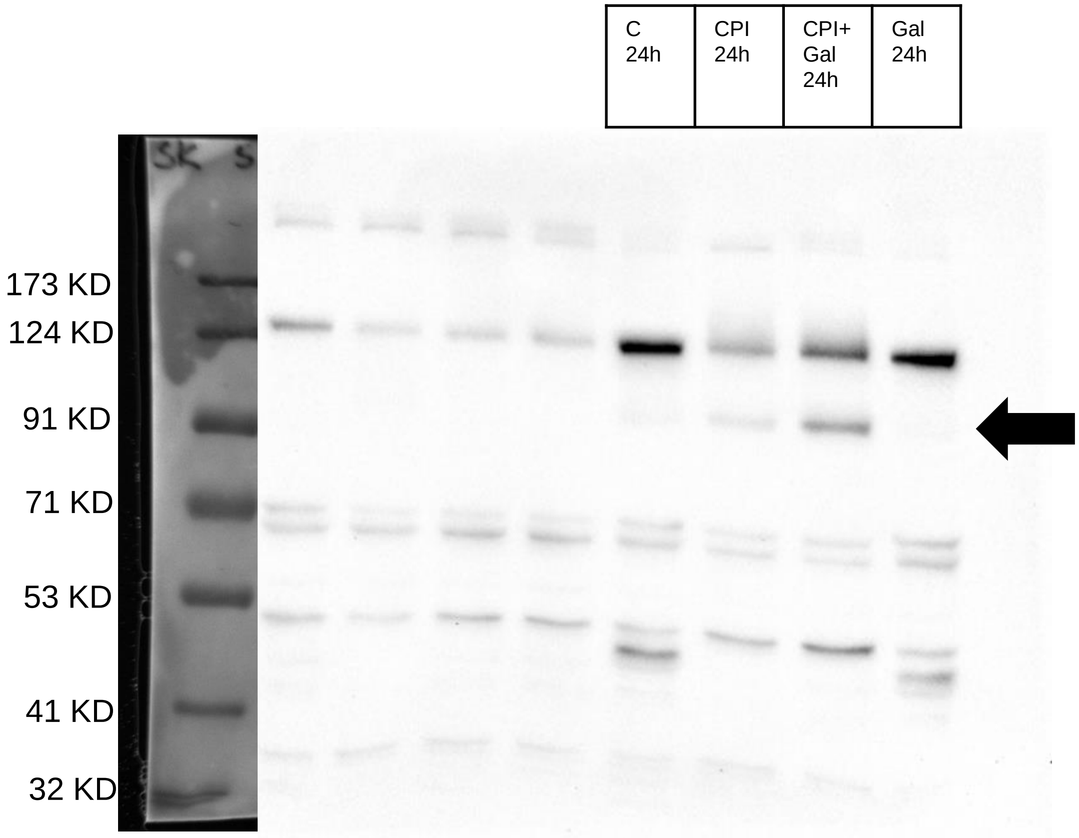

Cleaved parp 116 KD: 29.01.20, 25 µg Protein, 8% acrylamid gel, #9525 Cell Signaling Technology (1:1000), #7024 Cell Signaling Technology (1:20:000) , 200.000 seconds exposure time (Fig. 2E).

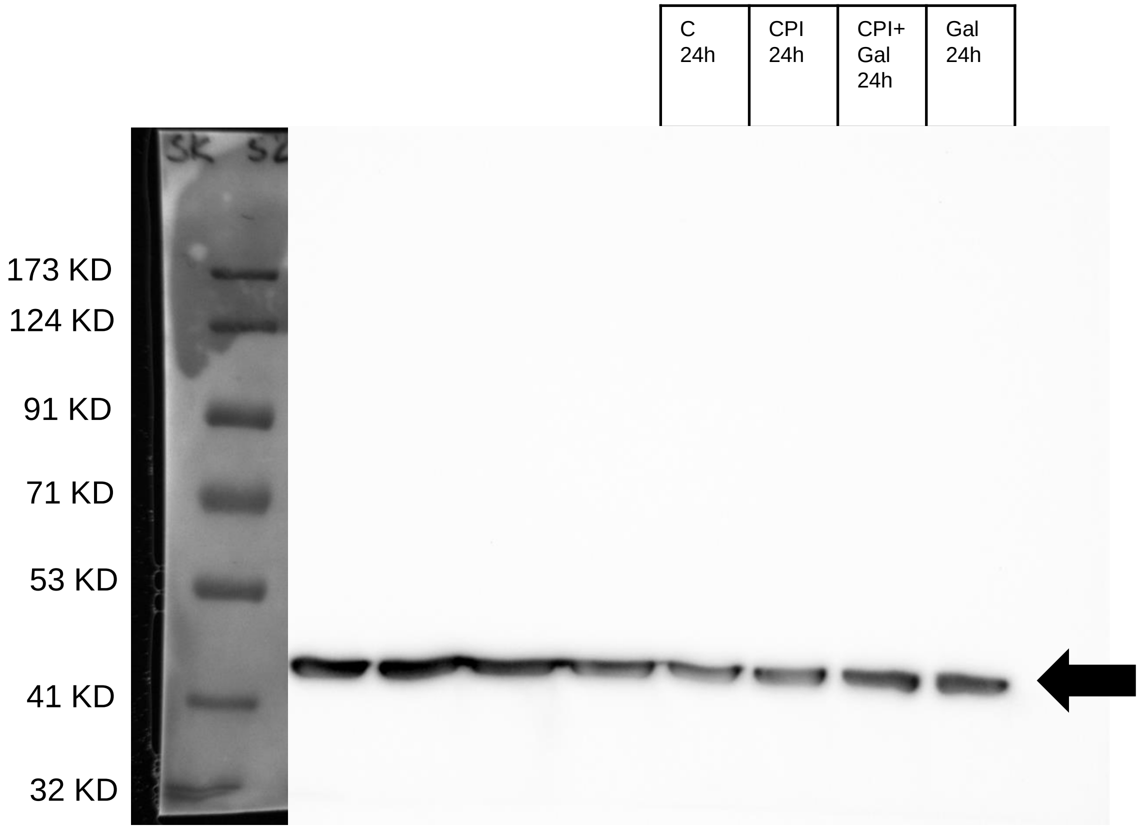

β-actin 42 KD: 30.01.20, 25 µg Protein, 8% acrylamid gel, A5441 Sigma-Aldrich, (1:20.000), 9044 Sigma-Aldrich (1:60:000), 50.000 seconds exposure time (Fig 2 E).

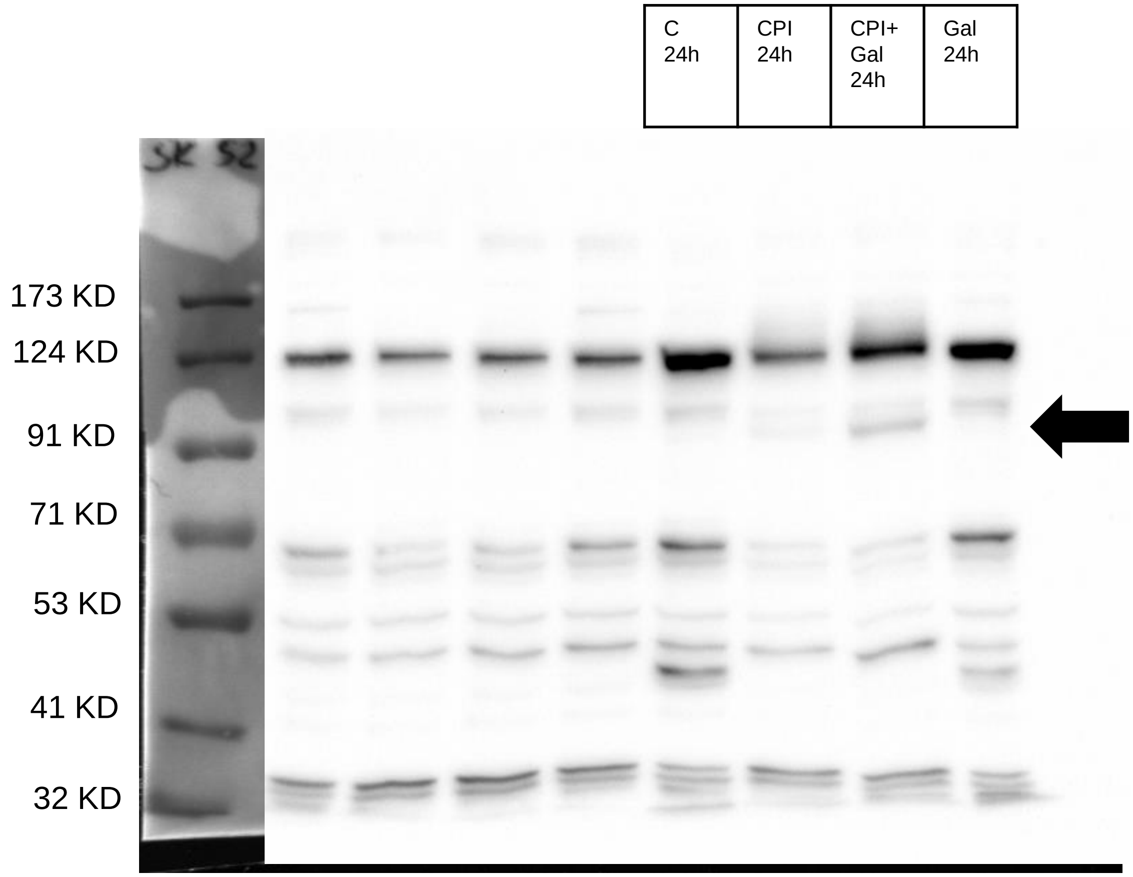

Cleaved parp 116 KD: 21.01.20, 25 µg Protein, 8% acrylamid gel, #9525 Cell Signaling Technology (1:1000), #7024 Cell Signaling Technology (1:20:000) , 200.000 seconds exposure time.

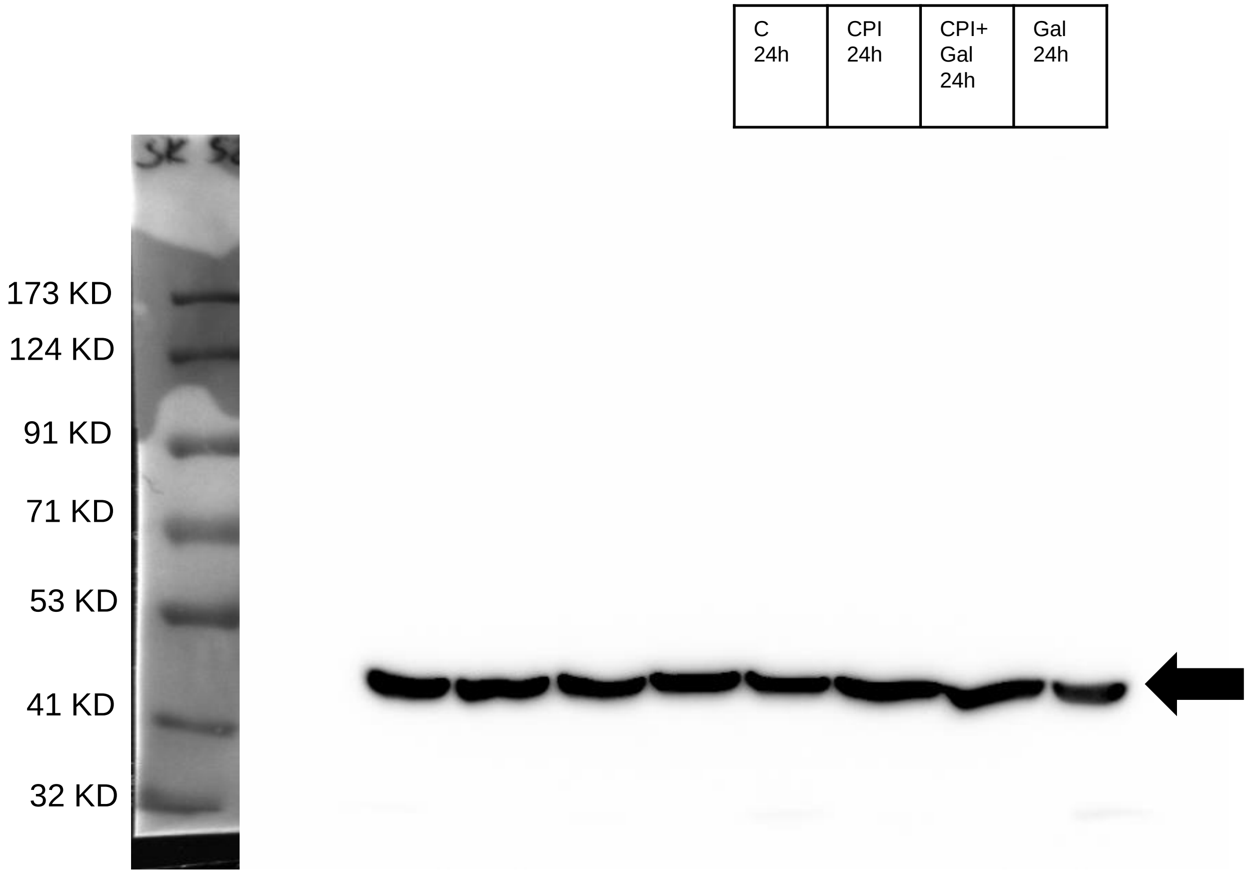

β-actin 42 KD: 22.01.20, 25 µg Protein, 8% acrylamid gel, A5441 Sigma-Aldrich, (1:20.000), 9044 Sigma-Aldrich (1:60:000), 50.000 seconds exposure time.

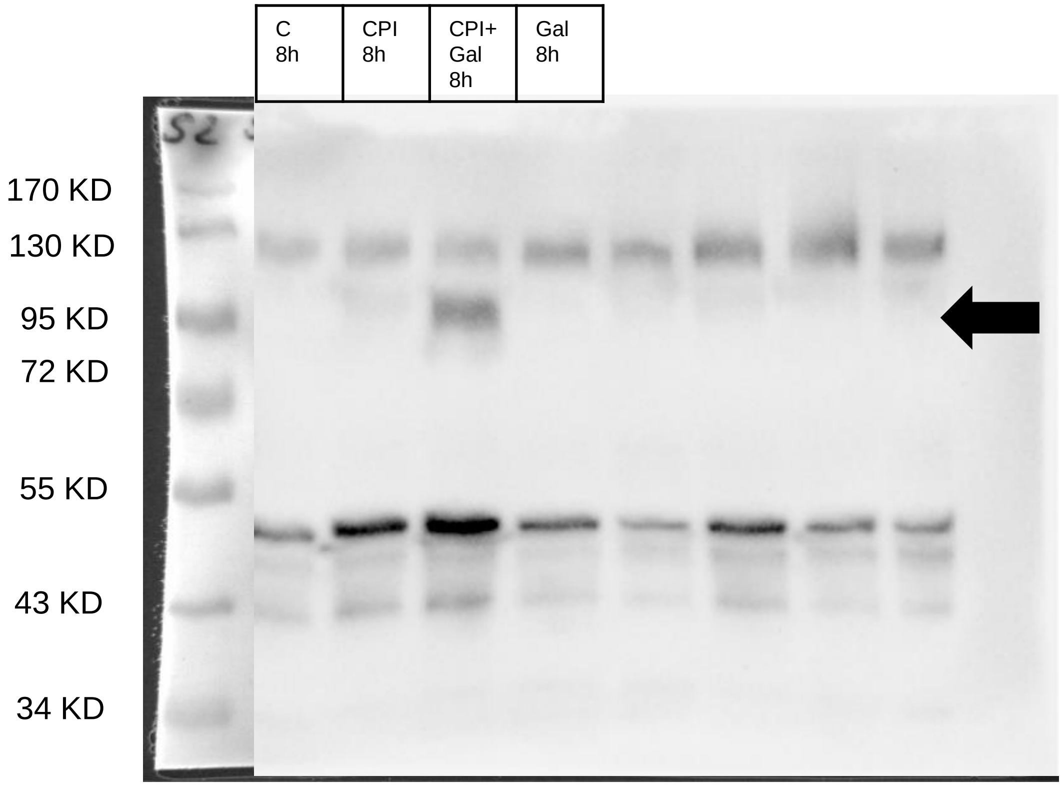

Cleaved parp 116 KD: 30.01.18, 40 µg Protein, 8% acrylamid gel, #9525 Cell Signaling Technology (1:1000), #7024 Cell Signaling Technology (1:20:000) , 200.000 seconds exposure time **(Fig 2E**).

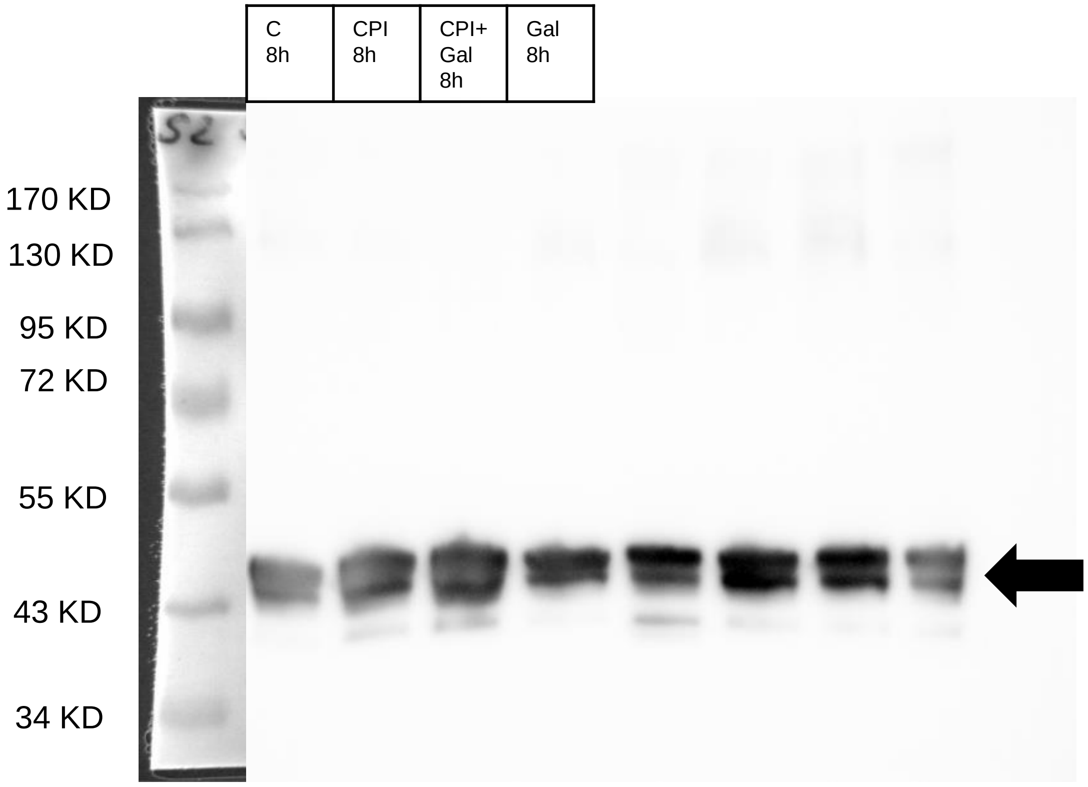

β-actin 42 KD: 01.02.18, 40 µg Protein, 8% acrylamid gel, A5441 Sigma-Aldrich, (1:20.000), 9044 Sigma-Aldrich (1:60:000), 100.000 seconds exposure time. (**Fig 2E)**

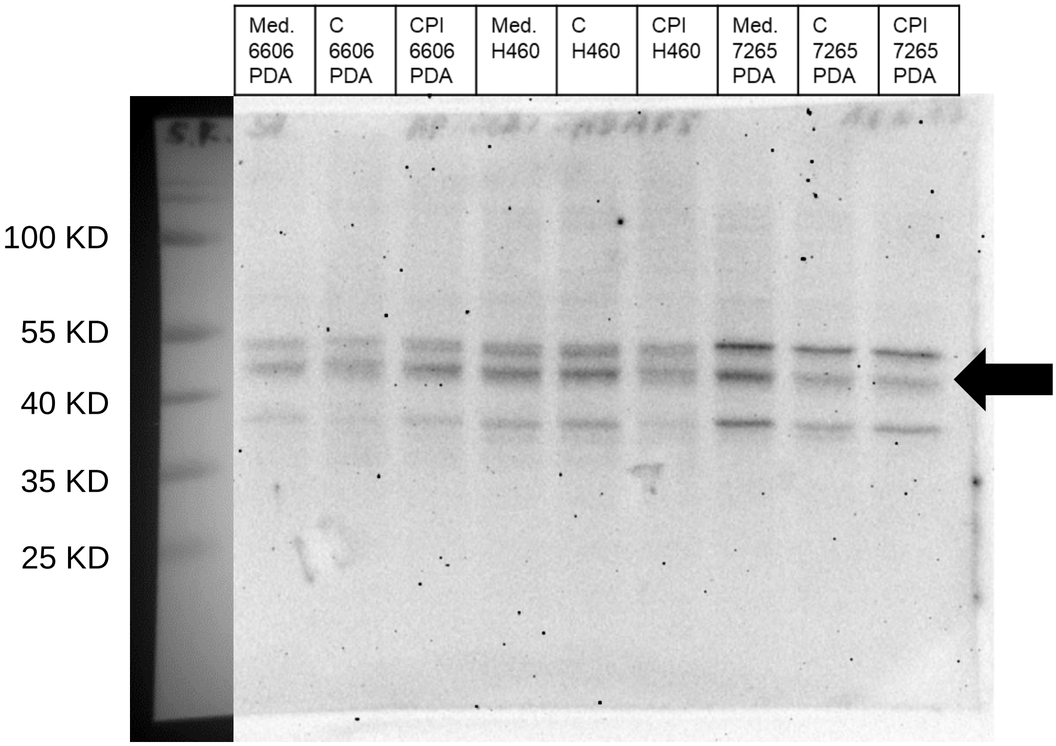

PDHA1 43 KD: 17.08.17, 10 µg Protein, 12% acrylamid gel, 9H9AF5 Thermo Fischer Scientific (1:1000), 9044 Sigma-Aldrich (1:60:000), 200.000 seconds exposure time (S3 Fig A).

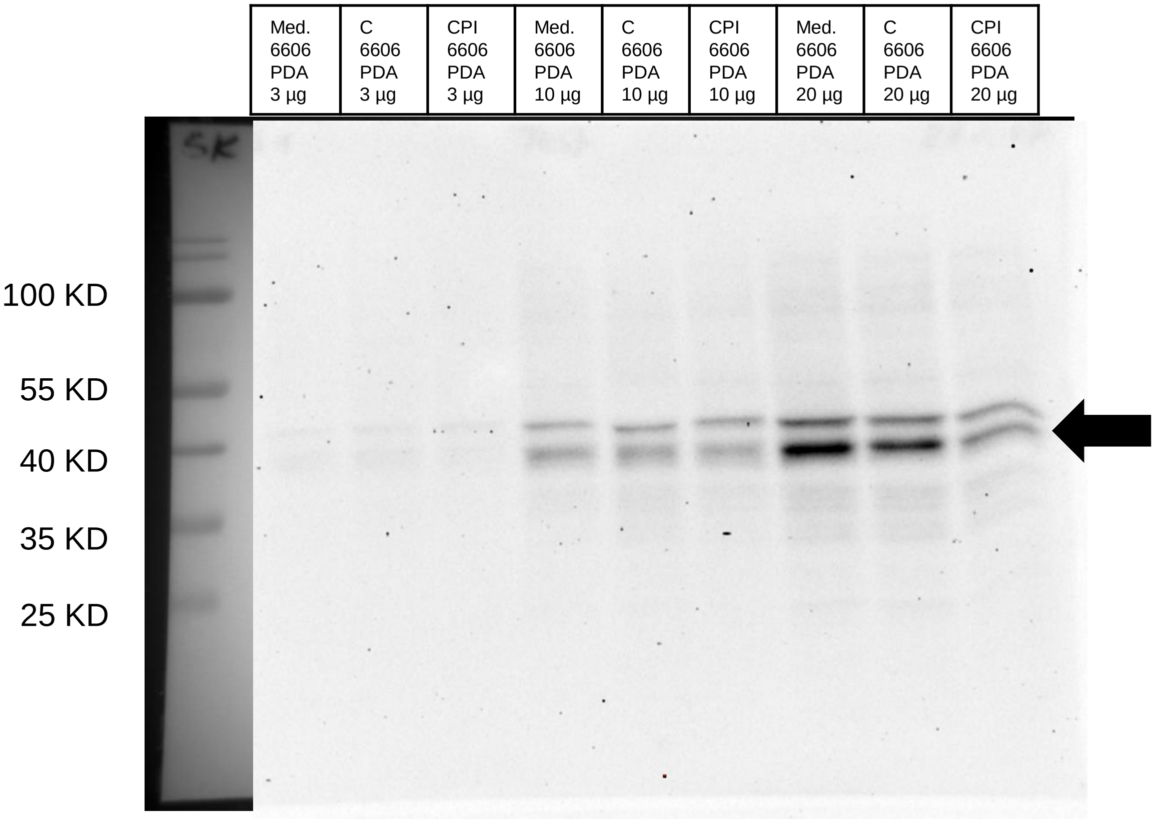

PDHA1 43 KD: 29.05.17, 3-20 µg Protein, 12% acrylamid gel, 9H9AF5 Thermo Fischer Scientific (1:1000), 9044 Sigma-Aldrich (1:60:000), 500.000 seconds exposure time.
